# Endothelial Cell Orientation and Polarity Are Controlled by Shear Stress and VEGF Through Distinct Signaling Pathways

**DOI:** 10.3389/fphys.2020.623769

**Published:** 2021-03-02

**Authors:** Anne-Clémence Vion, Tijana Perovic, Charlie Petit, Irene Hollfinger, Eireen Bartels-Klein, Emmanuelle Frampton, Emma Gordon, Lena Claesson-Welsh, Holger Gerhardt

**Affiliations:** ^1^Integrative Vascular Biology Laboratory, Max Delbruck Center for Molecular Medicine, Berlin, Germany; ^2^Université de Nantes, CNRS, INSERM, l’institut du thorax, Nantes, France; ^3^Institute for Molecular Bioscience, The University of Queensland, Brisbane, QLD, Australia; ^4^Beijer and Science for Life Laboratories, Rudbeck Laboratory, Department of Immunology, Genetics and Pathology, Uppsala University, Uppsala, Sweden; ^5^DZHK (German Center for Cardiovascular Research), Berlin, Germany; ^6^Berlin Institute of Health (BIH), Berlin, Germany

**Keywords:** endothelial cell, shear stress, VEGF, blood flow, signaling/signaling pathways

## Abstract

Vascular networks form, remodel and mature under the influence of multiple signals of mechanical or chemical nature. How endothelial cells read and interpret these signals, and how they integrate information when they are exposed to both simultaneously is poorly understood. Here, we show using flow-induced shear stress and VEGF-A treatment on endothelial cells *in vitro*, that the response to the magnitude of a mechanical stimulus is influenced by the concentration of a chemical stimulus, and vice versa. By combining different flow levels and different VEGF-A concentrations, front-rear polarity of endothelial cells against the flow direction was established in a flow and VEGF-A dose-response while their alignment with the flow displayed a biphasic response depending on the VEGF-A dose (perpendicular at physiological dose, aligned at no or pathological dose of VEGF-A). The effect of pharmaceutical inhibitors demonstrated that while VEGFR2 is essential for both polarity and orientation establishment in response to flow with and without VEGF-A, different downstream effectors were engaged depending on the presence of VEGF-A. Thus, Src family inhibition (c-Src, Yes, Fyn together) impaired alignment and polarity without VEGF-A while FAK inhibition modified polarity and alignment only when endothelial cells were exposed to VEGF-A. Studying endothelial cells in the aortas of VEGFR2^Y949F^ mutant mice and SRC^*iEC*–*KO*^ mice confirmed the role of VEGFR2 and specified the role of c-SRC *in vivo*. Endothelial cells of VEGFR2^Y949F^ mutant mice lost their polarity and alignment while endothelial cells from SRC^*iEC*–*KO*^ mice only showed reduced polarity. We propose here that VEGFR2 is a sensor able to integrate chemical and mechanical information simultaneously and that the underlying pathways and mechanisms activated will depend on the co-stimulation. Flow alone shifts VEGFR2 signaling toward a Src family pathway activation and a junctional effect (both *in vitro* and *in vivo*) while flow and VEGF-A together shift VEGFR2 signaling toward focal adhesion activation (*in vitro*) both modifying cell responses that govern orientation and polarity.

## Introduction

During embryonic development, all vertebrates initially establish a primitive network of vessels that subsequently remodels into a hierarchical vascular structure. This involves the creation of a primary vascular plexus that expands by sprouting angiogenesis (Isogai et al., 2003; Potente et al., 2011) followed by vascular remodeling to adapt vessel organization, shape and size; in its course, superfluous and inefficient segments are pruned away by active regression (Franco et al., 2015). The cellular and molecular regulation of this process is influenced by blood flow, hypoxia and metabolism. In this context, cells need to respond appropriately to both mechanical and chemical cues to ensure healthy tissue development and homeostasis.

Endothelial cells (ECs), which constitute the inner layer of vessels, are in particular under constant mechanical strains exerted by blood flow. Interestingly, ECs are able to sense small variations in the direction, magnitude, and regularity of blood flow–induced shear stress (Wang et al., 2013; Givens and Tzima, 2016) and respond to such changes by controlling their number, shape and movement (Culver and Dickinson, 2010; Baeyens et al., 2016a). Adaptation of ECs to flow is critical for both the development and the maintenance of a well-functioning cardiovascular system, as modification of capillary patterning allows for efficient oxygen and nutrient supply, while inward and outward remodeling of main arteries maintains appropriate blood pressure over the entire body (Baeyens et al., 2015). Physiological shear stress level, as found when the network is mature will favor EC elongation and orientation parallel to the flow direction. Furthermore, the Golgi of EC will position itself upstream of the nucleus, thus pointing against the flow direction (Franco et al., 2015) indicating their current migratory direction.

ECs are also well equipped to sense hypoxia. Hypoxic conditions drive the expression of vascular endothelial growth factors (VEGFs) by the surrounding tissues, which initiates endothelial sprouting through binding and activation of VEGF receptors (VEGFRs). Signaling downstream of these receptors is essential for vascular morphogenesis, as they control processes such as EC migration, proliferation and vessel permeability (Simons et al., 2016) and can influence arterial differentiation (Carmeliet and Tessier-Lavigne, 2005).

A long-standing question in developmental and cell biology relates to how cells integrate mechanical and chemical signals to orchestrate the morphogenic behaviors that ensure adequate tissue patterning. When looking at the receptors and signaling cascades implicated in both flow and chemical responses in ECs, it is clear that they are largely redundant and involve the same players (Jin et al., 2003; Koch and Claesson-Welsh, 2012). This suggests a cooperative or competitive action of chemical and mechanical stimuli during vascular bed formation, patterning, maturation and maintenance. In this context, VEGFR2 signaling is one of the most interesting examples. VEGFR2 is essential for VEGF-A-driven biological effects (Koch et al., 2011). It becomes activated and phosphorylated on tyrosine residues in response to VEGF-A: Y951, Y1059, Y1175, and Y1214 (human sequence numbers) (Matsumoto et al., 2005). The Y951 phospho-site (Y949 in mouse VEGFR2) presents a specific binding site for the T cell-specific adaptor which is implicated in VEGF-A-induced permeability, by regulating VEGFR2-dependent SRC signaling pathway at EC junctions (Sun et al., 2012). The Y1059 residues, located on the tyrosine kinase activation loop, are required for full kinase activity (Koch et al., 2011). The phosphorylated Y1175 (Y1173 in mouse VEGFR2) binds phospholipase Cg, which is of importance for endothelial extracellular-signal-regulated kinase ERK1/2 pathway activation (Takahashi et al., 2001). A phenylalanine knock-in mouse Vegfr2^Y1173F/Y1173F^ is embryonically lethal due to an arrest in EC development (Sakurai et al., 2005). Interestingly this phosphosite has also been described to be activated by flow, independently of VEGF, activating ERK1/2 and JNK pathways as well as eNOS (Chen et al., 1999; Jin et al., 2003). It has also been shown to activate NFκB both *in vitro* (Tzima et al., 2005; Coon et al., 2015) and *in vivo* (Baeyens et al., 2015). Finally, VEGFR2 Y1214 signaling induces activation of ERK1/2 and Akt pathways required for c-Myc-dependent gene regulation, endothelial proliferation, and vessel stability (Testini et al., 2019).

## Materials and Methods

### Mice and Treatments

The following mouse strains were used: VEGFR^Y949F^ mice (knock-in of phenylalanine (F) to replace the tyrosine (Y) at position 949 of VEGFR2 (Li et al., 2016) and c-Src-flox, Cdh5-CreERT2 mice designated as SRC^*iEC*–*KO*^ mice (Cdh5-CreERT2 mice were provided by Ralf Adams (MPI, Muünster, Germany) (Kogata et al., 2006; Wang et al., 2010). c-Src-floxed mice were delivered from the Nice Mice, National Resource Center for Mutant Mice, Model Animal Research Center, China) (Schimmel et al., 2020). Mice were maintained at the Uppsala University under standard husbandry conditions. All animal work was approved by the Uppsala University board of animal experimentation (permit 5.2.18-8927-16). To induce Cre-mediated deletion, tamoxifen (Sigma-Aldrich) was injected i.p. (100 μg) at P1, P2 and P3. Aortas were then collected at P6 onward. The investigators were blinded to genotype during experiments.

### Metatarsal Assay

Metatarsals were isolated from E16.5 mice using a protocol adapted from Song et al. (2015). After dissection, one metatarsal per well was placed in a μ-Plate 24 well ibiTreat plate with a 1.5 polymer coverslip (Ibidi) and left in 170 μl of MEM-alpha (Gibco) with 10% FCS and 1% penicillin/streptomycin (Sigma). After 3 days, media were replaced with 300 μl MEM-alpha + 10% FCS + 1% pen/strep per well and media changed every 48 h. To induce Cre activity, cells were treated with 1 μM of 4-hydroxytamoxifen (Sigma) after 5 days. After 14 days, metatarsals were fixed in 4% PFA in PBS for 20 min and antibodies were added in 3% Triton X-100, 1% Tween and 0.5% BSA in PBS. The following antibodies were used: GM130 (ref 560066, mouse, 1:500, BD Biosciences), ERG (ref ab92513, rabbit, 1:500, Abcam).

### Cell Culture and Microfluidic Chamber Experiments

HUVECs (passage 2–6; PromoCell) were routinely cultured in EBM-Bulletkit (Promocell). For flow experiments including static condition leading to WB lysate collection or immunofluorescence from Figures 2, 3, cells were cultured on 0.2% gelatin-coated slides (Menzel Glazer) and unidirectional laminar shear stress was applied using peristaltic pumps (Gilson) connected to a glass reservoir (ELLIPSE) and the chamber containing the slide. This device allows the circulation of 10 ml of medium on the slides, static slides has been exposed to 10 ml of medium without any medium circulation. For flow experiments under high shear stress (Figure 4) and immunofluorescence staining, cells were cultured on 0.2% gelatin-coated 0.4 ibidi slides (IBIDI) and unidirectional laminar shear stress was applied using the pumping system and control unit form IBIDI, allowing the circulation of 10 ml of medium as for the peristaltic system.

Local shear stress was calculated using Poiseuille’s law and averaged to 2 (Low SS) or 20 dyn/cm^2^ (High SS). For VEGF-A treatment, cells were exposed to shear stress for 24 h using EBM media (Promocell) without VEGF-A supplement and co-stimulated with 0, 0.5, 10 or 200, ng/ml of human VEGF-A165 (PeproTech ref 450-32) under the different flow conditions. For inhibition experiments, VEGFR2 inhibitors (SU1498, 1.5 μM; ZM323881, 4 nM), Src family inhibitor (SU6656, 500 nM), FAK inhibitor (PND-1186, 3 nM) and p38 inhibitor (SB203580, 1 μM) were added to the media 30 min prior flow start. Control condition were treated with DMSO diluted at 1/1,000 as all inhibitor used.

### Western Blotting

HUVECs were washed with cold PBS and scraped off in M-PER (Mammalian Protein Extraction Reagent; Thermo Fisher Scientific) completed protease and phosphatase inhibitors (Roche). Lysates were centrifuged and protein supernatant was quantified using the Lowry protein assay (Bio-Rad). Lysates were mixed with reducing sample buffer for electrophoresis and subsequently transferred onto polyvinylidene fluoride membranes. Equal loading was checked using Ponceau red solution. Membranes were incubated with primary antibodies (see below). After incubation with secondary antibodies (1:3,000; GE Healthcare), immunodetection was performed using an enhanced chemiluminescence kit (SuperSignal West Dura; Pierce), and bands were revealed using the Las-4000 imaging system. After initial immunodetection, membranes were stripped of antibodies, probed with total form of the phosphorylated form when suitable (PXL) and then reprobed with anti–GAPDH antibody. Values reported from Western blots were obtained by band density analysis using FIJI (ImageJ) and expressed as the ratio of the protein of interest to GAPDH or as the ratio of phosphorylated form to total form of the protein of interest. The following antibodies were used: GAPDH (ref MAB374, goat; 1:10,000; Millipore), VEGFR2 (ref 2479, rabbit, 1:1,000; cell signaling), p1175-VEGFR2 (ref 3770, rabbit, 1:1,000; cell signaling), p951-VEGFR2 (ref 2471, rabbit, 1:1,000; cell signaling), VE-cadherin (ref ab33168, rabbit, 1:1,000; abcam), p685-VE-cadherin (ref ab119785, rabbit, 1:1,000; abcam), ZO-1(ref 61-7300, rabbit, 1:1,000; Thermo Fisher Scientific), FAK (ref 3285, rabbit, 1:1,000; cell signaling), Claudin5 (ref 34-1600, rabbit, 1:1,000; Invitrogen), Paxillin (ref 610051, rabbit, 1:1,000; BD Biosciences), p118-Paxillin (ref 2541, rabbit, 1:1,000; cell signaling).

### Immunofluorescence Staining

HUVECs were fixed with 4% PFA in PBS for 10 min at room temperature (RT). Blocking/permeabilization was performed using blocking buffer consisting of 5% BSA (Sigma-Aldrich), 0.5% Triton X-100 (Sigma-Aldrich), 0.01% sodium deoxycholate (Sigma-Aldrich), and 0.02% sodium azide (Sigma-Aldrich) in PBS at pH 7.4 for 45 min at RT. Primary antibodies were incubated at the desired concentration in 1:1 Blocking buffer/PBS at RT for 2 h and secondary antibodies were incubated at the desired concentration in 1:1 blocking buffer/PBS for 1 h at RT. Aortas were fixed with 4% PFA in PBS overnight at 4°C. Blocking/permeabilization was performed using blocking buffer consisting of 1% FBS, 3% BSA (Sigma-Aldrich), 0.5% Triton X-100 (Sigma-Aldrich), 0.01% sodium deoxycholate (Sigma-Aldrich), and 0.02% sodium azide (Sigma-Aldrich) in PBS at pH 7.4 for 1 h at RT. Primary antibodies were incubated at the desired concentration in 1:1 Blocking buffer/PBS overnight at 4°C and secondary antibodies were incubated at the desired concentration in 1:1 blocking buffer/PBS for 2 h at RT. DAPI (Sigma-Aldrich, 1/10,000, 5 min) was used for nuclear labeling. Cells and aortas were mounted in Mowiol. The following antibodies were used *in vitro*: VE-cadherin (ref sc-6458, goat; 1:100; Santa cruz), ZO-1 (ref 61-7300, rabbit, 1:500; Thermo Fisher Scientific), GM130 (ref 610822, mouse, 1:1,000;BD Biosciences). The following antibodies were used *in vivo*: GOLPH4 (ref ab28049, rabbit; 1:500; Abcam), VE–cadherin (ref 555289; rat; 1/100; BD Biosciences).

### Microscope Image Acquisition

Images from fluorescently labeled HUVECs were acquired using a LSM 700 upright microscope equipped with a Plan-Apochromat 20×/0.8 NA Ph2 objective. Images were taken at room temperature using Zen 2.3 software. Bright-field images were taken using a Leica DMIL LED microscope equipped with a 10×/0.22 NA Ph1 objective and a CCD camera (DFC3000 G). Images were acquired at room temperature while the cells were still in their culture medium using LAS X software (Leica). Images of aortas were taken using a LSM 780 inverted microscope (Zeiss) equipped with a Plan-Apochromat 20×/0.8 NA Ph2 objective or with a Plan-Apochromat 63×/1.4 NA DIC objective. The microscope was equipped with a photon multiplier tube detector. Images were taken at room temperature using Zen 2.3 software (Zeiss). Images of metatarsals were acquired using a LSM 710 inverted microscope equipped with Plan-Apochromat 10×/0.45 NA and 20×/0.8 NA objectives. For all animal experiments, the investigators were unaware of the genotypes of the animals while acquiring images.

### Cell Junction Activity Analysis

Cell junction morphology analysis was done in HUVECs stained for VE-cadherin using the patch and classified Matlab code previously developed by Bentley et al. (2014) adapted for 2D images. Two status were defined: activated vs. stabilized, and divided into 3 level: from low to high. Activated junctions were defined as serrated or reticular and stabilized as straight thick junctions. Each image taken was divided into small pieces of images allowing to visualized only a portion of the junction and these small images were presented blindly and randomly to the user who then classified the junction accordingly. This technic ensured an unbiased analysis of the junctions regarding the treatment and regarding the global shape of the cell.

### Flow-Induced Orientation Analysis

To analyze the orientation of cells, we calculated the angle formed between the vector of the flow direction (obtained by knowledge of flow direction within the slide) and the “orientation vector” given by orientation of the main axe of each cell. Each value for each cell is then used to plot the hemi-roses presented in Figure 2A. To plot the bar graphs presented in Figures 2B, 4B, 5B, 6B the cells were classified in 2 categories: aligned with the flow direction, and not aligned with the flow direction. For *in vitro* experiment, aligned with the flow was defined as an absolute value of angle between 0 and 45°, not aligned with the flow as an absolute value of angle between 45 and 90°; for *in vivo* experiments, aligned with the flow was defined as an absolute value of angle between 0 and 15° and not aligned with the flow as an absolute value of angle between 15 and 90°. Angle calculation and roses presentation was done automatically using a homemade Matlab script validated on the first experiment by a comparison to hand calculation with FIJI.

### Flow-Induced Polarity Analysis

To analyze the polarization of cells, we calculated the angle formed between the vector of the flow direction (obtained by knowledge of flow direction within the slide) and the “golgi vector” given by the line from the center of the nucleus to the center of the Golgi. Of note, static experiments are not exposed to flow and therefor calculation has been made using an arbitrary direction given by the geometry of the ibidi slide. Each value for each cell is then used to plot the roses presented in Figure 2C. To plot the bar graphs presented in Figures 2D, 4D, 5D, 6D, the cells were classified in 3 categories: against the flow direction, sided to the flow direction and with the flow direction. For *in vitro* experiment, with the flow was defined as an absolute value of angle between 0 and 45°, sided as an absolute value of angle between 45 and 135° and against the flow as an absolute value of angle between 135 and 180°; for *in vivo* experiment with the flow was defined as an absolute value of angle between 0 and 30°, sided as an absolute value of angle between 30 and 150° and against the flow as an absolute value of angle between 150 and 180°. Angle calculation and roses presentation was done automatically using a homemade Matlab script validated on the first experiment by a comparison to hand calculation with FIJI. Metatarsal polarity analysis was performed using FIJI with values defined as for *in vivo* experimental values (with: 0–30°, sided: 30–150°, and against: 150–180°).

### Statistical Analysis

Statistical analysis was performed using GraphPad Prism software. For *in vitro* and *in vivo* experiments, two-way ANOVA (data distribution was assumed to be normal) were used, followed by a Tukey test or a Fisher LSD test when conditions were considered experimentally independent. Details of the statistical test used for each experiment can be found in the figure legends. The investigators were blinded to genotype during experiments and quantification.

## Experimental Approach

**FIGURE 1:**
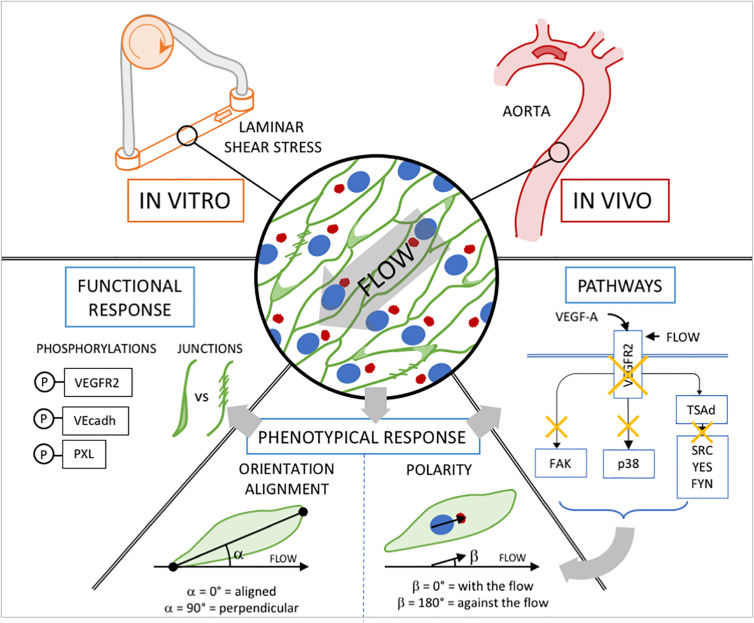
Experimental approach. *In vitro*, We subjected ECs to either static condition, low or high shear stress (SS, 2 or 20 dyn/cm^2^ for 24 h) and to different VEGF-A concentrations ranging from physiological (0.5 and 10ng/mL) to pathological (200 ng/mL) levels. *In vivo*, we used the linear part of aorta which is exposed to high SS and free from VEGF-A influence. **Phenotypical response:** Cell orientation/alignment refers to the long axis of ECs as they adopt their prototypical elongated cell shape under the influence of blood flow *in vivo*, or equivalent shear stress induced by medium’s flow *in vitro*. Determining the center of mass of the golgi in relation to the center of mass of the cell nucleus delivers a vector used as a proxy for front-rear polarity of migrating cells. **Functional response:** biochemical analysis of VEGFR2 phosphorylation on Tyrosine 1175 and 951; VE-cadherin phosphorylation on Tyrosine 685; Paxillin phosphorylation on Tyrosine 118. classification of junction type through VE-cadherin staining. **Pathways:** inhibition through drug treatment (*in vitro*) or Knock-Out strategies (*in vivo*) and analysis of the phenotypical response.

To study how physical forces and growth factors jointly influence front-rear polarity and cell elongation toward the flow direction (alignment/orientation), we subjected ECs to either static conditions, low or high shear stress (SS, 2 or 20 dyn/cm^2^ for 24 h) and to different VEGF-A concentrations ranging from physiological (0.5 and 10 ng/mL) to pathological (200 ng/mL) levels. Cell orientation refers to the long axis of ECs as they adopt their prototypical elongated cell shape under the influence of blood flow *in vivo*, or equivalent shear stress induced by medium flow *in vitro*. Alignment in this context depicts whether this long-axis of the ECs is aligned with the direction of the flow. Similarly, front-rear polarity is also assessed in relation to the flow direction. Unlike elongation or alignment, the polarity vector assigns a front and a rear to the cell, in a way that is normally associated with dynamic movement of cells in a certain direction. When they migrate, many cell types, including ECs, position their centrosome and Golgi ahead of the nucleus in the direction of migration. Therefore, determining the center of mass of the Golgi in relation to the center of mass of the cell nucleus delivers a vector that can be used as a proxy for front-rear polarity of migrating cells. Recent work illustrated that ECs under flow *in vivo* and *in vitro* follow the same principle as they orientate and migrate against the direction of flow during vascular remodeling (Kupfer et al., 1982; Franco et al., 2015). We therefore determined the Golgi-nucleus vector to establish front-rear polarity under all conditions of SS and growth factor stimulation. Throughout the analysis below, cell alignment and polarity in relation to the direction of flow will serve as reference system for the phenotypical flow response of ECs (Figure 1).

As the polarized movement of ECs requires junctional rearrangements and turnover, both during angiogenic sprouting and vascular remodeling under flow (Koch et al., 2011; Conway et al., 2013; Neto et al., 2018), we also assessed endothelial junctional patterns under the various conditions. Previous studies classified junctional features that correlate with junctional dynamics and VE-cadherin turnover (Bentley et al., 2014), providing a useful quantitative framework for the assessment of flow and growth factor effects. We used this classification tool adapted for a 2D layer to evaluate junctional activity in our different *in vitro* conditions. We complemented these results with biochemical analysis of VE-cadherin phosphorylation on Tyrosine 685, which contributes to VE-cadherin internalization (Orsenigo et al., 2012). Since VEGFR2 is a flow sensor (Tzima et al., 2005) when associated to PECAM and VE-cadherin, in addition to its VEGF-A receptor activity, we then employed biochemical analysis to study its activation. We exposed ECs to selective inhibitor treatments to identify pathway activity patterns and their relevance for the different aspects of the endothelial phenotypical response to flow. Finally, we validated our results using an *in vivo* approach. The aorta is exposed to high SS (similar level as used in our *in vitro* experiments) in its linear, thoracic part and free from VEGF-A influence. In this model, ECs forming the linear part are highly elongated and aligned with the flow direction and mostly polarized against the flow. In contrast EC display rounded shape and random alignment in the aortic arch exposed to turbulent flow generating low SS (Gimbrone and García-Cardeña, 2013). We took advantage of these aortic characteristics to assess EC alignment in deficient mice models to validate our *in vitro* finding on phenotypical response to flow (Figure 1).

## Results

### Combination of Flow Exposure and VEGF-A Treatment Modifies Endothelial Cell Orientation and Polarity

**FIGURE 2:**
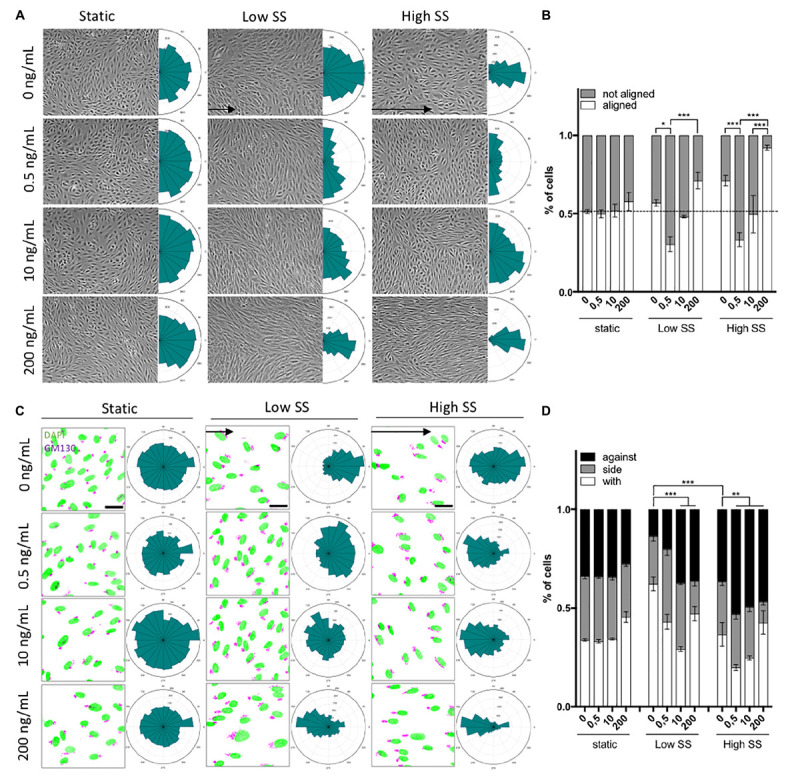
Dose-dependent effect of shear stress and VEGF-A concentration on cell orientation and polarity. **(A)** Representative picture (phase contrast) of endothelial cells exposed to shear stress for 24 h (static: 0 dyn/cm^2^; Low SS: 3 dyn/cm^2^; High SS: 20 dyn/cm^2^) and associated orientation quantification shown as circular plots (*N* = 3; between 1,500 and 3,000 cells analyzed). **(B)** Quantification of percentage of cells aligned with the flow direction (in between 45° around the flow axis, *N* = 3, between 1,500 and 3,000 cells analyzed), Data presented as Mean + SEM. **(C)** Representative picture (Immunofluorescence) of endothelial cells exposed to shear stress for 24 h (Low SS: 3 dyn/cm^2^; High SS: 20 dyn/cm^2^) (scale bars: 30 μm). **(D)** Quantification of golgi position around the nucleus compared to the flow direction (*N* = 3; with: in between 0 and 45° around the flow axis, side: 45–135°, against: 135–180°; between 1,500 and 3,000 cells analyzed). Two-way ANOVA; Tukey’s *post hoc*, *p < 0.05; **p < 0.01; ***p < 0.001.

As VEGF-A induces proliferation of ECs (Koch et al., 2011), we first controlled whether our experimental condition influenced cell density and therefore the analyses. In all conditions, static, low SS (2 dyn/cm2), or high SS (20 dyn/cm^2^), VEGF-A (0.5–200 ng/ml) did not affect the EC number (Supplementary Figure 1A). Both high and low SS exposure reduced the number of cells to about 75% of that in the static conditions. Alignment analyses showed that the ECs main axis in static condition (no flow, 0 dyn/cm^2^) was randomly distributed, with no effect of VEGF-A addition (Figures 2A,B). The only effect observed was an increase in cell elongation after treating ECs with 200 ng/mL of VEGF-A (Supplementary Figure 1B).

Low SS (2 dyn/cm^2^, no VEGF-A) had no effect on cell alignment, which remained random. VEGF-A addition (0.5 ng/ml) to low SS promoted alignment perpendicular to the flow direction while treatment with 200 ng/ml VEGF-A promoted EC alignment with the flow (Figures 2A,B). This showed that the effect of VEGF-A under low SS was biphasic, dependent on the dose used.

High SS itself (20 dyn/cm^2^, no VEGF-A) enhanced alignment with the flow. VEGF-A addition (0.5 ng/ml) to high SS made ECs align perpendicular to the flow direction. At high VEGF-A concentrations (200 ng/ml), alignment with the flow was increased, as observed under low SS (Figures 2A,B) but even more pronounced.

When assessing polarity, ECs in static culture remained randomly polarized in the absence and presence of VEGF-A (Figures 2C,D). At low SS in the absence of VEGF-A, ECs polarized in the direction “with” the flow, meaning that their Golgi was mostly placed behind the nucleus compared to the flow direction. On the contrary, high SS triggered a partial polarization against the flow. In both low and high SS, VEGF-A promoted polarity against the flow, in a dose-response manner: the higher the VEGF-A concentration, the better cells polarized against the flow (Figures 2C,D).

Taken together, these data show that ECs align and orient themselves relative to flow by integrating chemical and flow conditions and that the magnitude of the stimuli direct the response. With the more pronounced combined stimulation, ECs aligned efficiently and polarized against the flow.

### Combination of Flow Exposure and VEGF-A Treatment Affects VEGFR2 Phosphorylation, Adherens Junction Stability and Focal Adhesion Activity

We then looked at cell adherens junctions. Under static conditions, VE-cadherin staining and quantification of stabilized (straight) and activated (serrated) junctions did not show any effect of VEGF-A treatment after 24 h (Supplementary Figures 2A,B). ZO-1 appeared localized at the junction for all VEGF-A concentration (Supplementary Figure 2A). Some discontinuities in the adherens junctions were observed at 200 ng/mL (Supplementary Figure 2, red arrow). This was not associated to a change in VE-cadherin and ZO-1 protein levels upon SS and VEGF-A exposure (Supplementary Figures 3B,C).

**FIGURE 3:**
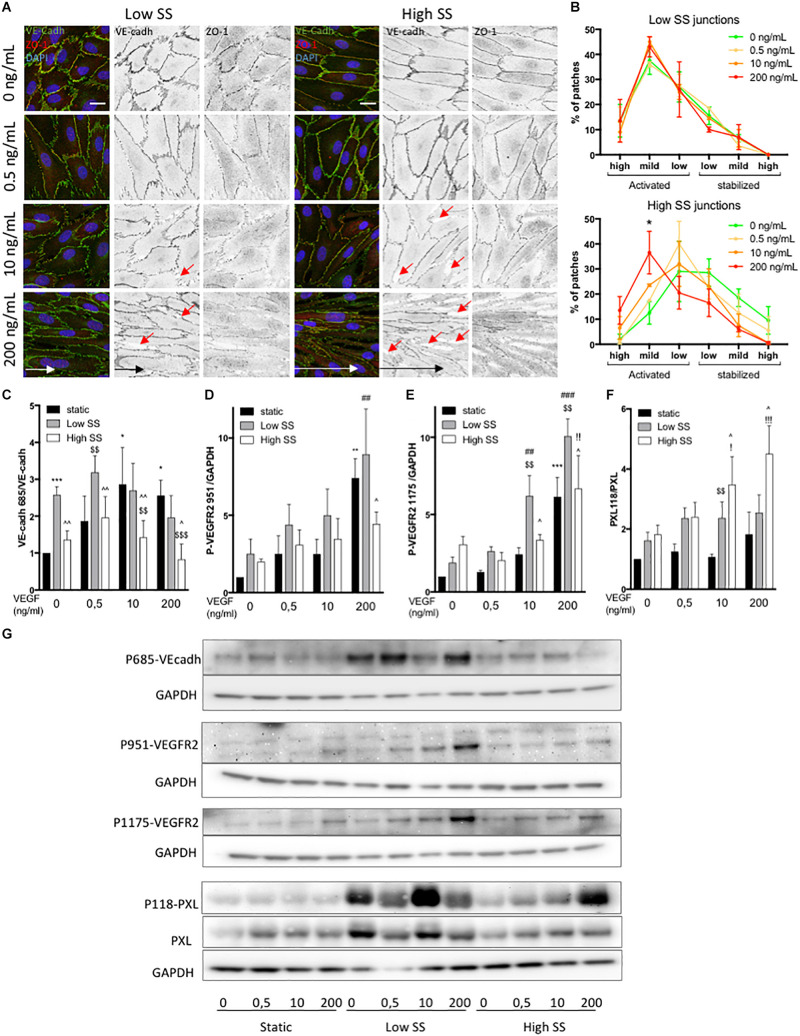
Effect of flow and VEGF-A combination on VEGFR2, junctional and focal adhesion activity. **(A)** Representative picture (Immunofluorescence) of endothelial cells exposed to shear stress for 24 h (Low SS: 3 dyn/cm^2^; High SS: 20 dyn/cm^2^). Red arrows indicate gaps in the ECs monolayer **(B)** Quantification of junction status based on their morphology (*N* = 3; 100 patches analyzed blinded by images, 5–8 images per N) (scale bars: 20 μm, same magnification for all images). **(C)** WB analysis of VE-cadherin phosphorylation, *N* = 5, **(D)** WB analysis of VEGFR2 phosphorylation on Tyr951. *N* = 4 **(E)** WB analysis of VEGFR2 phosphorylation on Tyr1175. *N* = 4 **(F)** WB analysis of Paxilllin phosphorylation on Tyr118. *N* = 4 **(G)** WB representative images. Data presented as Mean + SEM. Two-way ANOVA; Fisher LSD test, *p < 0.05; **p < 0.01; ***p < 0.001 compared to static 0 VEGF; ^##^*p* < 0.01; ^###^*p* < 0.001 (compared to LSS 0 VEGF); ^!!^*p* < 0.01 (compared to HSS 0 VEGF); ^$$^*p* < 0.01; ^[*dollar*][*dollar*][*dollar*]^*p* < 0.001 compared to static from the same VEGF concentration; ^∧∧^*p* < 0.05; ^∧∧∧^*p* < 0.01 compared to Low SS from the same VEGF concentration.

Upon low SS alone (no VEGF-A), ECs showed a high level of activated/serrated junctions (Figures 3A,B). VEGF-A treatment had no additional effect on junction activation which remained high. In contrast, high SS induced stabilization/straightening of the junctions and increasing the concentration of VEGF-A correlated with an increase in serrated VE-cadherin junctions (Figures 3A,B). Junctional ZO-1 was higher in ECs exposed to high SS than in ECs exposed to low SS (Figure 3A). VEGF-A treatment induced a loss of this junctional ZO-1 both under low and high SS (Figure 2A). Additionally, both under low and high SS, high VEGF-A concentrations (10 and 200 ng/mL) led to loosening of the adherens junctions, which displayed increased number of gaps in the endothelial layer (indicated as red arrows on Figure 3A), to a higher extent at high SS.

To better understand the junctional modifications, we analyzed VE-cadherin phosphorylation on Tyrosine 685, which contributes to VE-cadherin internalization (Orsenigo et al., 2012). Under static condition, VEGF-A treatment increased VEcadh^Y685^ level in a dose dependent manner (Figures 3C,G). ECs exposed to low SS have high VE-cadherin phosphorylation level independently of VEGF-A concentration (Figures 3C,G), matching the observation of high level of serrated junction in this flow condition. VEcadh^Y685^ levels remained low under high SS independently of VEGF-A concentration and despite increasing serrated junctions.

Since VEGFR2 is a flow sensor (Tzima et al., 2005) when associated to PECAM and VE-cadherin in addition to its VEGF-A receptor activity, we evaluated its phosphorylation status in our conditions. First, we confirmed that both tyrosine 1175 and 951 were phosphorylated in a dose-response manner upon VEGF-A treatment under static conditions (Figures 3D,E,G). VEGFR2 expression was stable in static condition but significantly increased in response to flow in the absence of VEGF-A (Supplementary Figure 3A). Of note, high VEGF-A treatment (200 ng/mL) slightly but significantly decreased VEGFR2 expression, suggesting modification in protein turn over. VEGFR2^Y1175^ phosphorylation level was increased by SS (Figures 3E,G) in the absence of VEGF-A, with high SS inducing more VEGFR2^Y1175^ phosphorylation than low SS. In comparison, VEGFR2^Y951^ phosphorylation was increased by SS but at an equivalent level between low and high SS (Figures 3D,G). When ECs were exposed to low SS, VEGF-A treatment strongly increased VEGFR2^Y951^ and VEGFR2^Y1175^ phosphorylation in a dose-response manner while this effect was not present (VEGFR2^Y951^) or mild (VEGFR2^Y1175^) when ECs were exposed to high SS (Figures 3D,G).

As VEGFR2 activation can also affect focal adhesion formation (Pietilä et al., 2019), we then checked the expression levels of FAK and Paxillin and the phosphorylation level of Paxillin on tyrosine 118, which controls focal adhesion turn over, affinity to FAK and cell migration (López-Colomé et al., 2017). FAK and Paxillin displayed stable expression over all of the tested conditions (Figure 3F and Supplementary Figure 3F). Paxillin^Y118^ phosphorylation was activated by both low and high SS in the absence of VEGF-A compared to static condition (Figures 3F,G). At low SS, increasing VEGF-A concentration did not significantly change Paxillin^Y118^ phosphorylation level. In contrast, at high SS, VEGF-A treatment increased it in a dose-response manner (Figures 3F,G).

Combined, these data show that low flow induced an “activated” junctional morphology and phosphorylation of VEGFR2 and VE-cadherin; these effects were further augmented by VEGF-A. In contrast, high flow combined with VEGF-A preferentially induced focal adhesion signaling.

### VEGFR2 Is Involved in Orientation and Polarity Responses Through Different Signaling Cascades

In order to understand better how activation of VEGFR2 contributed to focal adhesion or adherens junction turn-over in our setting and what was the role of each one in orientation and polarity, respectively, we exposed ECs to pharmacological inhibitors targeting the known pathways downstream of VEGFR2 activation. We decided to focus on the condition that displayed the clearest dichotomic effects of flow and VEGF-A on polarity and alignment respectively, namely high SS with or without 10 ng/mL of VEGF-A.

**FIGURE 4:**
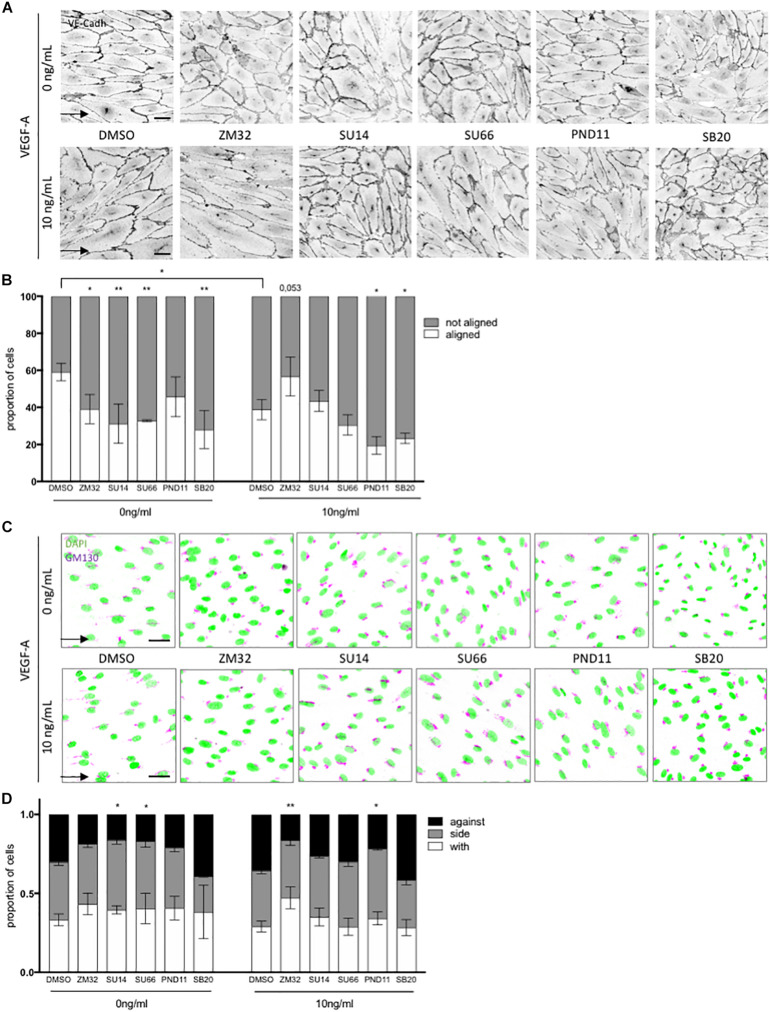
Pathways involved in flow and VEGF responses. **(A)** Representative picture of junction (VE-cadherin mentioned as VE-cadh) of endothelial cells exposed to shear stress for 24 h (High SS: 20 dyn/cm^2^) without or with inhibitors (scale bars: 40 μm, same magnification for all images). **(B)** Quantification of percentage of cells aligned with the flow direction (in between 30° around the flow axis. DMSO, *N* = 5, inhibitors *N* = 3). **(C)** Representative picture polarity of endothelial cells exposed to shear stress for 24 h (High SS: 20 dyn/cm^2^) without or with inhibitors (scale bars: 40 μm, same magnification for all images). **(D)** Quantification of golgi position around the nucleus compared to the flow direction (DMSO, *N* = 5, inhibitors *N* = 3. with: in between 0 and 45∘ around the flow axis, side: 45–135°, against: 135–180°). Arrow represents flow direction (High SS). Two-way ANOVA; *p < 0.05; **p < 0.01; ***p < 0.001. VEGFR2 inhibitors (SU1498, 1.5 mM; ZM323881, 4 nM), Src family inhibitor (SU6656, 500 nM), FAK inhibitor (PND-1186, 3 nM) and p38 inhibitor (SB203580, 1 mM) were added to the media 30 min prior flow start.

VEGFR2 inhibition by SU1498 and ZM323881 (two different kinases inhibitor with a high selectivity for VEGFR2 but targeting different downstream effectors) had a strong effect on cell elongation and alignment with the flow in absence of VEGF-A; ECs were less elongated (Supplementary Figure 4) and lost their alignment with the flow (Figures 4A,B). Surprisingly, SU1498 had no significant effect on cell alignment and elongation in presence of VEGF-A (Figures 4A,B and Supplementary Figure 4) and ZM323881 tended to restore alignment with the flow in presence of VEGF-A. VEGFR2 inhibition also impaired ECs polarization against the flow both without (SU1498) and with VEGF-A (ZM323881) (Figures 4C,D).

We then inhibited SRC family kinases members (SU6656), FAK (PND-1186) and p38 (SB203580) in order to target pathways responsible for ECs cellular processes implicated in adhesion and migration. SRC family kinases inhibition significantly reduced ECs alignment with the flow and polarity against the flow in the absence of VEGF-A but had no effect in presence of VEGF-A. In contrast, FAK inhibition significantly impaired alignment with the flow and polarity against the flow only when ECs were exposed to VEGF-A (Figures 3A–D) but not when VEGF-A was absent. This demonstrated that cell orientation and polarity were under the control of different pathways in the presence or absence of VEGF-A. Interestingly, p38 inhibition which prevents stress activation and migration of ECs, blocked EC alignment and elongation both with and without VEGF-A, but had no effect on cell polarity; ECs remained partially polarized against the flow as in control condition (Figures 4A–D and Supplementary Figure 4).

These data support the notion that alignment in response to flow is dependent on VEGFR2 and that alignment and polarity are differently regulated by pathways downstream of VEGFR2.

### VEGFR2 and SRC Control Endothelial Cell Orientation and Polarity *in vivo* in Matures Arteries

**FIGURE 5:**
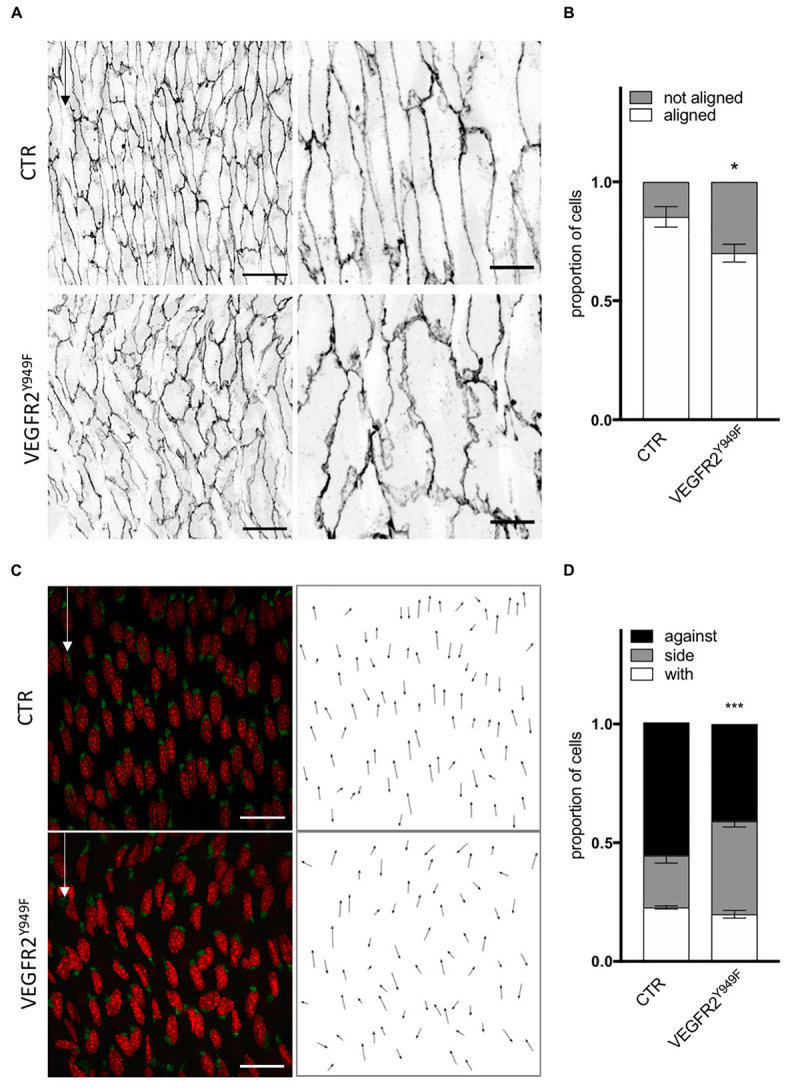
VEGFR2 mutation impaired cell orientation and cell polarity *in vivo*. **(A)** Representative images of VE-cadherin staining of endothelial cell from the aortas of littermate P6 pups (CTR) or carrying VEGFR2 mutation (Y949F) (scale bars: 25 and 10 μm, respectively). **(B)** Quantification of cells aligned with the flow direction (in between 15° around the flow axe, *N* = 5, Data presented as Mean + SEM. **(C)** Representative images of golgi staining of endothelial cell from the aortas of P6 pups littermate (CTR) or carrying VEGFR2 mutation (Y949F) (scale bars: 25 μm). **(D)** Quantification of golgi position around the nucleus compared to the flow direction (*N* = 5; with: in between 0 and 30° around the flow axe, side: 30–150°, against: 150–180°). Two-way ANOVA; Tukey’s *post hoc*, *p < 0.05; ***p < 0.001.

As SRC family kinases inhibition impaired ECs response to flow in the absence of VEGF-A *in vitro*, we evaluated the relevance of the VEGFR2—SRC pathway *in vivo*. First, As VEGFR2-949 phosphorylation by VEGF stimulation recruits activated Src to EC junctions to phosphorylate VE-cadherin, we analyzed the aortas of VEGFR2^Y949F^ mutant mice. We observed that ECs from the aorta of VEGFR2^Y949F^ mutant mice lost their alignment in response to flow (Figure 5A) and had a reduced cell length (Supplementary Figures 5A,B), similar to what we observed *in vitro* following treatment with the VEGFR2 inhibitor ZM323881 (Supplementary Figure 4). Loss of VEGFR2 phosphorylation on this specific site also impaired polarity of ECs against the flow; the proportion of ECs well polarized against the flow was decreased compared to control mice (Figure 5C). To study whether this effect was associated specifically with SRC and not YES or FYN *in vivo*, we assessed alignment and polarity of ECs in aortas of mice lacking endothelial SRC (SRC^*iEC*–*KO*^ mice).

**FIGURE 6:**
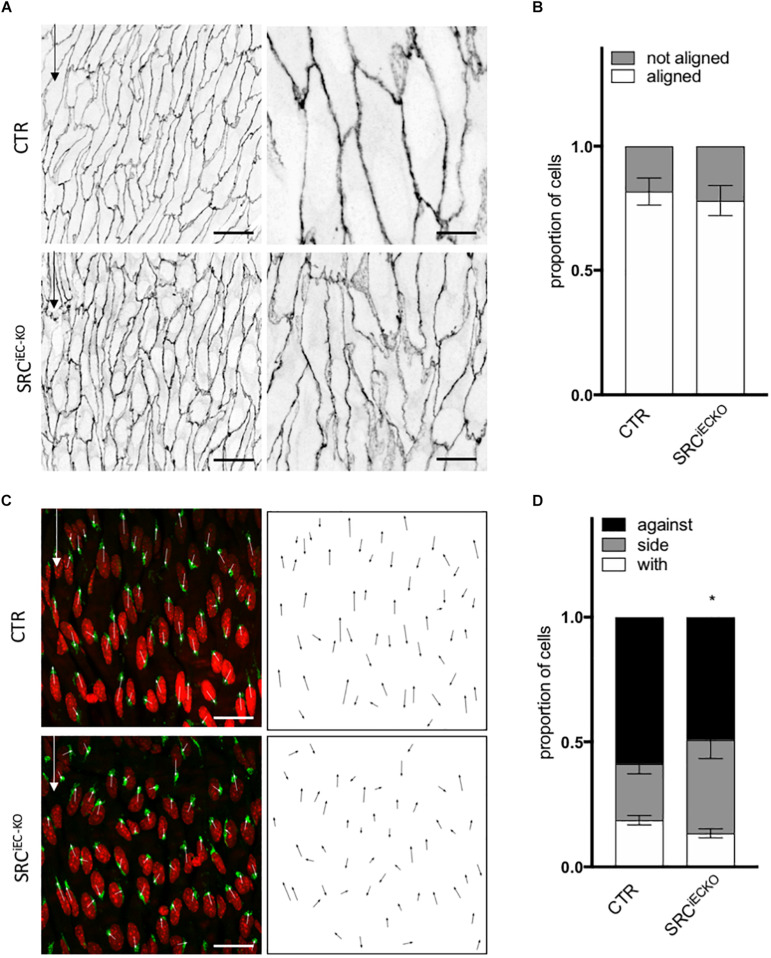
SRC deletion in ECs mutation impaired cell orientation and cell polarity *in vivo*. **(A)** Representative images of VE-cadherin staining of endothelial cell from the aortas of P6 pups littermate (CTR) or deleted for SRC in ECs (SRC^*iEC*– *KO*^) (scale bars: 25 and 10 μm, respectively). **(B)** Quantification of cells aligned with the flow direction (in between 15° around the flow axe, WT, *N* = 6; KO, *N* = 7, Data presented as Mean + SEM. **(C)** Representative images of golgi staining of endothelial cell from the aortas of P6 pups littermate (CTR) or deleted for SRC in ECs (SRC^*iEC*– *KO*^) (scale bars: 25 μm). **(D)** Quantification of golgi position around the nucleus compared to the flow direction (WT, *N* = 6; KO, *N* = 7; with: in between 0 and 30° around the flow axe, side: 30–150°, against: 150–180°). Two-way ANOVA; Tukey’s *post hoc*, *p < 0.05; ***p < 0.001.

Cell shape and junction morphology was similarly affected in SRC^*iEC*–*KO*^ aortas as in VEGFR2^Y949F^aortas. Surprisingly, however, EC alignment with the flow was not impaired in SRC^*iEC*–*KO*^ aortas (Figures 6A,B) nor was there any effect on EC length (Supplementary Figure 5B). Nevertheless, polarity against the flow was reduced in SRC^*iEC*–*KO*^ mice compared to control (Figures 6C,D) similarly to VEGFR2^Y949F^ mutant mice. Finally, to confirm the flow specificity of these observations, we used an *ex vivo* sprouting assay from mouse metatarsals (Song et al., 2015; Schimmel et al., 2020), which reproduced angiogenesis in a “no flow” but high growth factor condition. Polarity of ECs at the tip position was not altered in metatarsal explants from SRC^*iEC*–*KO*^ mice compared to control mice (Supplementary Figure 6). These results indicate that SRC activity is not generally required for ECs to polarize, but selectively involved in flow induced cell polarity.

In conclusion, the *in vitro* and *in vivo* data agree on the importance of VEGFR2, specifically through pY951 signaling, in endothelial cell alignment and polarity. Moreover, the Src pathway regulates polarity but not alignment of ECs.

## Discussion

In the past decade, many efforts have been made to elucidate how mechanical forces and chemical signals contribute to vascular formation and patterning. Several mechano-sensory pathways controlling cell shape (Levesque and Nerem, 1985; Wojciak-Stothard and Ridley, 2003; Noria et al., 2004), polarity (Franco et al., 2015; Kwon et al., 2016) and migration (Baeyens et al., 2016b; Rochon et al., 2016) during vascular patterning have been described and the basic cellular and molecular mechanisms controlling angiogenesis have been well characterized (Potente et al., 2011; Potente and Mäkinen, 2017). Nevertheless, how ECs integrate signals coming from both mechanical and chemical stimuli at the same time is not well understood, despite its importance in physiology and pathology. Here, we observed that these signals can have synergistic or antagonistic effects depending on the feature observed.

We first confirm that VEGF-A treatment increases cell elongation, VEGFR2^Y1175^, VEGFR2^Y951^ and VEcadh^Y685^ phosphorylation levels in a dose dependent manner under static condition, as expected from the literature (Simons et al., 2016; Cao et al., 2017). We also observe already described effects of low SS and high SS on cell alignment (Chien, 2007; Wang et al., 2013), junctions aspect (Chiu and Chien, 2011) and changes in VEGFR2^Y1175^ phosphorylation (Jin et al., 2003) in the absence of VEGF-A. However, beyond these observations, we also identify new features associated with the combination of flow and VEGF-A. Polarity against the flow is established in a dose-response to VEGF-A both under low and high SS, but only high SS could trigger polarity against the flow without VEGF-A. Furthermore, alignment displays a biphasic response depending on the VEGF-A level (aligned without VEGF or at pathologically high dose of VEGF-A, but perpendicular to flow at physiological levels of VEGF-A). This indicates that orientation and polarity are controlled by different mechanisms. Interestingly, the effect of VEGF-A on polarity under high SS was seen even from the lowest concentrations used (0.5 ng/mL) while a higher concentration (10 ng/ml) was required to reach the same percentage of polarity against the flow at low SS. This suggests that both parameters mutually control the cells’ sensitivity to the other. Shear Stress modifies the sensitivity to VEGF-A, but VEGF-A in turn also affect the cell’s ability to respond to flow.

When inhibiting VEGFR2, the effects of both SS and VEGF-A on alignment and polarity are lost suggesting that VEGFR2 could be the hub controlling these responses. The effect of SU1498 on polarity was significant only in the absence of VEGF-A, whereas ZM323881 significantly affected polarity only with VEGF-A. Studies using these inhibitors highlight their selectivity for VEGFR2 but also show that SU1498 prevents ERK1/2 signaling cascade (Boguslawski et al., 2004) while ZM323881 inhibits rather p38 and Rac1 pathways (Whittles et al., 2002; Garrett et al., 2007). Altogether this suggests that these inhibitors might differently restrict pathways downstream of VEGFR2 that contribute to polarity establishment with or without VEGF-A.

Inhibiting downstream effectors of VEGFR2, namely FAK and SRC family members, uncovered that the orientation and polarity of ECs are controlled differently in the presence or absence of VEGF-A. Without VEGF-A, alignment with the flow and polarity against the flow are dependent on SRC family activity while in the presence of VEGF-A, alignment and polarity against the flow are dependent on FAK activity. Interestingly, p38 inhibition only impaired alignment with the flow, not polarity which remains mostly against the flow. In this context, p38 inhibition suggests that ECs can establish and modify their planar polarity without changing their cell shape.

These conclusions are further supported by our *in vivo* analysis, as VEGFR2 phosphorylation at Y951 contributes to ECs alignment with the flow and polarity against the flow in the absence of VEGF-A. By genetically deleting SRC specifically in ECs, we highlight that SRC is essential for polarity control but not for alignment, although this was expected from our inhibitor experiments *in vitro*. Interestingly, the inhibitor used *in vitro* has a strong affinity toward Yes and Fyn, the two other SRC family members. While Yes and Fyn are structurally highly similar to SRC, evidence for their distinct roles in the endothelium are currently emerging (Eliceiri et al., 1999; Gordon et al., 2016; Schimmel et al., 2020). Combining our *in vivo* and *in vitro* results, we can propose that loss of SRC impairs endothelial polarity against the flow, which is not compensated by Fyn or Yes. Loss of SRC had no effect on ECs alignment *in vivo* suggesting that this feature could be under the control of Yes or Fyn. Further work will need to establish the exact contribution of Yes and Fyn to the alignment of ECs in response to flow. In the same line as for the p38 inhibition *in vitro*, we highlight that polarity and alignment can occur independently, but here showing that change in cell shape does not required planar polarity establishment.

By pointing their Golgi better against the flow at high SS, ECs display a cellular organization that is characteristic for their migration against the flow. In both SS conditions, VEGF-A will cause an activation that will enable ECs to adjust their junctions and basal adhesions more dynamically and polarize better against the flow even if exposed to a lower unidirectional force. Whether polarity always correlates with cell displacement (migration), however, remains unclear. Two hypotheses arise from the increased polarity against the flow; the first one is that ECs are indeed migrating better against the flow at high SS or with VEGF-A addition, the second would be that while ECs polarize against the flow at high SS or with VEGF-A, they migrate very little due to high counteracting apical forces.

Both hypotheses raise the question of tension sensing and balance between basal and lateral forces. Force transmission occurs through different structures in ECs (Campinho et al., 2020; Gordon et al., 2020); cell-cell junctions and cell adhesions to the matrix have been both well described as mechano-sensitive elements. In our study, the structural changes of VE-cadherin junctions do not correlate with alteration of VEcadh^Y685^ phosphorylation, suggesting that other junctional players are involved. Interestingly ZO-1 has been shown to control endothelial cell-cell tension and its loss, while loosening tension in-between cells favors focal adhesion formation (Tornavaca et al., 2015) and therefore reinforces basal adhesion. In our setting, ZO-1 localization at junctions is specifically decreased by VEGF-A when ECs are exposed to SS compared to static condition and could be the missing player explaining the visual modifications of adherens junctions. Additionally, increased Paxillin phosphorylation also correlates with the loss of junctional ZO-1. Together with our observation, this supports our hypothesis that tension forces in between ECs could be highly different in between our different *in vitro* conditions. VEGF-A addition under SS, would trigger loss of cell-cell tension (lateral) while increasing cell-matrix tension (basal). This effect of VEGF-A appears to be more pronounced at high SS compared to low SS.

Another situation where ECs adapt their shape and polarity is when they migrate to close a wound. In such a case, driven by the first row of cells directly in contact with the free edge, ECs polarize collectively and migrate toward the wound. In that situation, the origin of the signal is different from a flow situation. In a wound assay, most cells are not exposed to the free edge and receive an indirect cue for migration through force transmission via the lateral junctions. Recent work from Carvalho et al. (2019) shows that by decreasing VE-cadherin tension (therefore lateral forces) in between cells, ECs failed to collectively polarize toward the wound. Under flow, every cell is independently exposed to the same directional signal: SS at their apical side. Combining these facts and observations allows to hypothesize that the origin of the mechanical signal and its way of transmission in-between cells is crucial to determine if loosening of the lateral tension will lead to loss or reinforcement of polarity. In the case of a wound closure, loosening junctions decreases collective polarity toward the wound because ECs distant from the wound become blind to its location. In our settings, loosening junctions increases polarity against the flow because ECs become more capable of detecting flow direction as signal from the lateral junctions does not interfere with the apical cue each cell perceive.

Interestingly, while testing inhibitor effects on polarity and alignment under high SS, we find that SRC family inhibition is efficient only without VEGF-A, i.e., a situation in which ZO-1 is present at the junctions and phospho-Paxillin is low, lateral tension should be high and basal tension low. In contrast, FAK inhibition is efficient only with VEGF-A, a situation in which ZO-1 is delocalized from the junctions and phospho-Paxillin is high, suggesting low lateral tension and high basal tension. Therefore, it is tempting to speculate that rather than having different pathways controlling polarity and alignment independently, the same pathways could be in charge of polarity and alignment, but their relative contribution would vary depending on the presence or absence of VEGF-A. SRC family members would participate when VEGF-A is absent and FAK pathways would take over once VEGF-A is present to loosen the junction.

Finally, modification in flow sensitivity in ECs has been ascribed to the mechanosensitive complex formed by VEGFRs-PECAM-VE-cadherin (Baeyens et al., 2015), and in particular to the ratio of VEGFR3 or VEGFR2 engaged in this complex, thus modifying at which range of SS ECs align with the flow. Here we show that the joint presence of flow and VEGF-A can also act as a lever to influence ECs alignment and polarity mostly through changes in the balance of the different VEGFR2 phospho-sites that become activated. Whether or not alignment and polarity go precisely through the same type of complex and if VEGFR3 could also play a role will need to be demonstrated.

## Data Availability Statement

The raw data supporting the conclusions of this article will be made available by the authors, without undue reservation, to any qualified research.

## Ethics Statement

The animal study was reviewed and approved by the Uppsala University board of animal experimentation (permit 5.2.18-8927-16).

## Author Contributions

A-CV designed the study, performed experiments, analyzed the results, and wrote the manuscript. TP and CP performed the experiments and analyzed the results. EB-K, IH, and EF performed the experiments. EG performed the experiments and reviewed the manuscript. LC-W provided the mice, designed the *in vivo* study, and reviewed the manuscript. HG designed the study and wrote the manuscript. All authors contributed to the article and approved the submitted version.

## Conflict of Interest

The authors declare that the research was conducted in the absence of any commercial or financial relationships that could be construed as a potential conflict of interest.

## Supplementary Material

Supplementary Figure 1 VEGF-A treatment does not affect cell number but increases cell elongation. **(A)** Quantification of endothelial cells number under flow and VEGF-A. **(B)** Quantification of aspect ratio (length of main axis/length of short axis) of endothelial cells under flow and VEGF-A. *N* = 3, between 1,500 and 3,000 cells analyzed. *N* = 3, around 1,500 cells analyzed. Data presented as Mean + SEM. Two-way ANOVA; Tukey’s *post hoc*, ^∗∗∗^*p* < 0.001 compared to static 0 VEGF; ^###^*p* < 0.001 (compared to LSS 0 VEGF); ^!!!^*p* < 0.001 (compared to HSS 0 VEGF).

Supplementary Figure 2 VEGF treatment does not change junctions activation under static condition. **(A)** Representative picture (Immunofluorescence) of endothelial cells exposed to VEGF-A for 24 h. Red arrows indicate gaps in the ECs monolayer **(B)** Quantification of junction status based on their morphology (*N* = 3; 100 patches analyzed blinded by images, 5–8 images per N).

Supplementary Figure 3 Proteins expression upon flow and VEGF treatment. **(A)** VEGFR2 expression assessed by WB, *N* = 6. **(B)** ZO1 expression assessed by WB, *N* = 3. **(C)** VE-cadherin expression assessed by WB, *N* = 5. **(D)** FAK expression assessed by WB, *N* = 5. ANOVA followed by Tukey *post Hoc*; ^∗^*p* < 0.05; ^∗∗^*p* < 0.01. **(E)** Representative pictures of the quantified WB.

Supplementary Figure 4 Inhibitors effect on cell elongation. Quantification of aspect ratio (length of main axis/length of short axis) of endothelial cells under high SS with or without VEGF-A (10 ng/mL) and with or without inhibitors (DMSO, *N* = 5, inhibitors *N* = 3) One-way ANOVA; Tukey’s *post hoc*, ^∗∗∗^*p* < 0.001 compared to DMSO 0 VEGF; ^###^*p* < 0.001 compared to DMSO 10 VEGF.

Supplementary Figure 5 VEGFR2 mutation impairs cell length but not SRC deletion *in vivo*. **(A)** Quantification of ECs length in the aortas of P6 pups littermate (CTR) or carrying VEGFR2 mutation (Y949F). *N* = 5 **(B)** Distribution of cell length. *N* = 5 **(C)** Quantification of ECs length in the aortas of P6 pups littermate (CTR) or deleted for SRC in ECs (SRC^*iEC*–*KO*^). *N* = 3–5 **(D)** Distribution of cell length. Unpaired *T*-Test; ^∗∗∗^*p* < 0.001.

Supplementary Figure 6 ECs polarity is not impaired during directional sprouting upon loss of c-Src. Representative images and quantification of polarity of ECs sprouting out of metatarsal *ex vivo*. With means that ECs polarize in the direction of the sprout (0–30°); side, ECs present their golgi on the side of the cell (30–150°); against, ECs polarize in the opposite direction of the sprout (150–180°). *N* = 294 cells analyzed from 12 metatarsals from 2 independent experiments.

Supplementary Table 1 *p*-Values for each comparison for Figures 2B,D. Two-way ANOVA; Tukey’s *post hoc*, ^∗^*p* < 0.05; ^∗∗^*p* < 0.01; ^∗∗∗^*p* < 0.001. Gray lines: statistics presented on the graphs. Bold: significant difference.

Supplementary Table 2 *p*-Values for each comparison for Figures 3C–F. Two-way ANOVA; Fisher LSD. ^∗^*p* < 0.05; ^∗∗^*p* < 0.01; ^∗∗∗^*p* < 0.001. Gray lines: statistics presented on the graphs. Bold: significant difference.
