# Supplementary figures and images for: Endothelial Cell Orientation and Polarity Are Controlled by Shear Stress and VEGF Through Distinct Signaling Pathways
Source: Front Physiol. 2021 Mar 2;11:623769. doi: 10.3389/fphys.2020.623769 (PMC7960671; doi:10.3389/fphys.2020.623769)

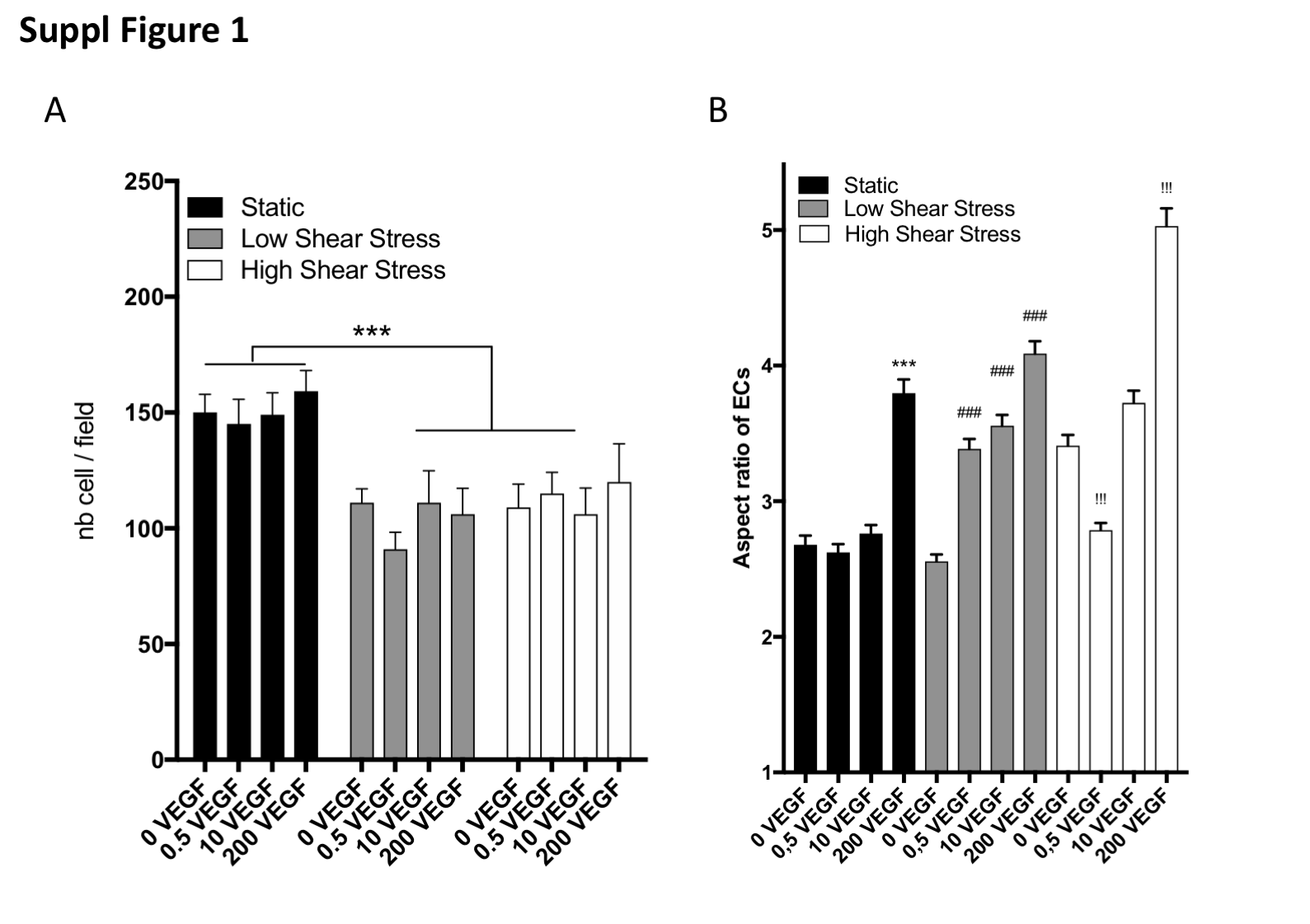

Supplement: Supplementary Figure 1 — VEGF-A treatment does not affect cell number but increases cell elongation. (A) Quantification of endothelial cells number under flow and VEGF-A. (B) Quantification of aspect ratio (length of main axis/length of short axis) of endothelial cells under flow and VEGF-A. N = 3, between 1,500 and 3,000 cells analyzed. N = 3, around 1,500 cells analyzed. Data presented as Mean + SEM. Two-way ANOVA; Tukey’s post hoc, ∗∗∗p < 0.001 compared to static 0 VEGF; ###p < 0.001 (compared to LSS 0 VEGF); !!!p < 0.001 (compared to HSS 0 VEGF). [file Image_1.TIFF]

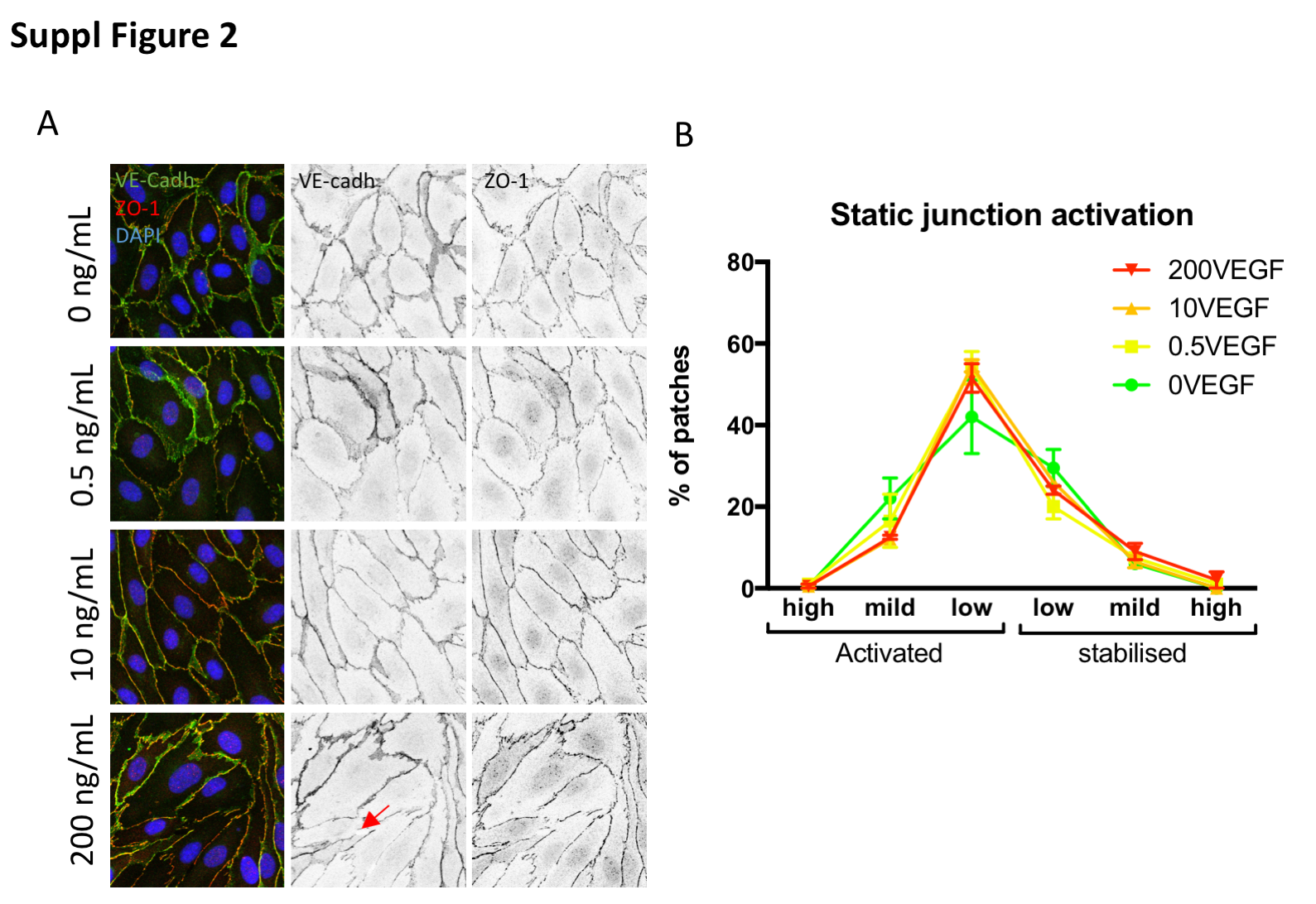

Supplement: Supplementary Figure 2 — VEGF treatment does not change junctions activation under static condition. (A) Representative picture (Immunofluorescence) of endothelial cells exposed to VEGF-A for 24 h. Red arrows indicate gaps in the ECs monolayer (B) Quantification of junction status based on their morphology (N = 3; 100 patches analyzed blinded by images, 5–8 images per N). [file Image_2.TIFF]

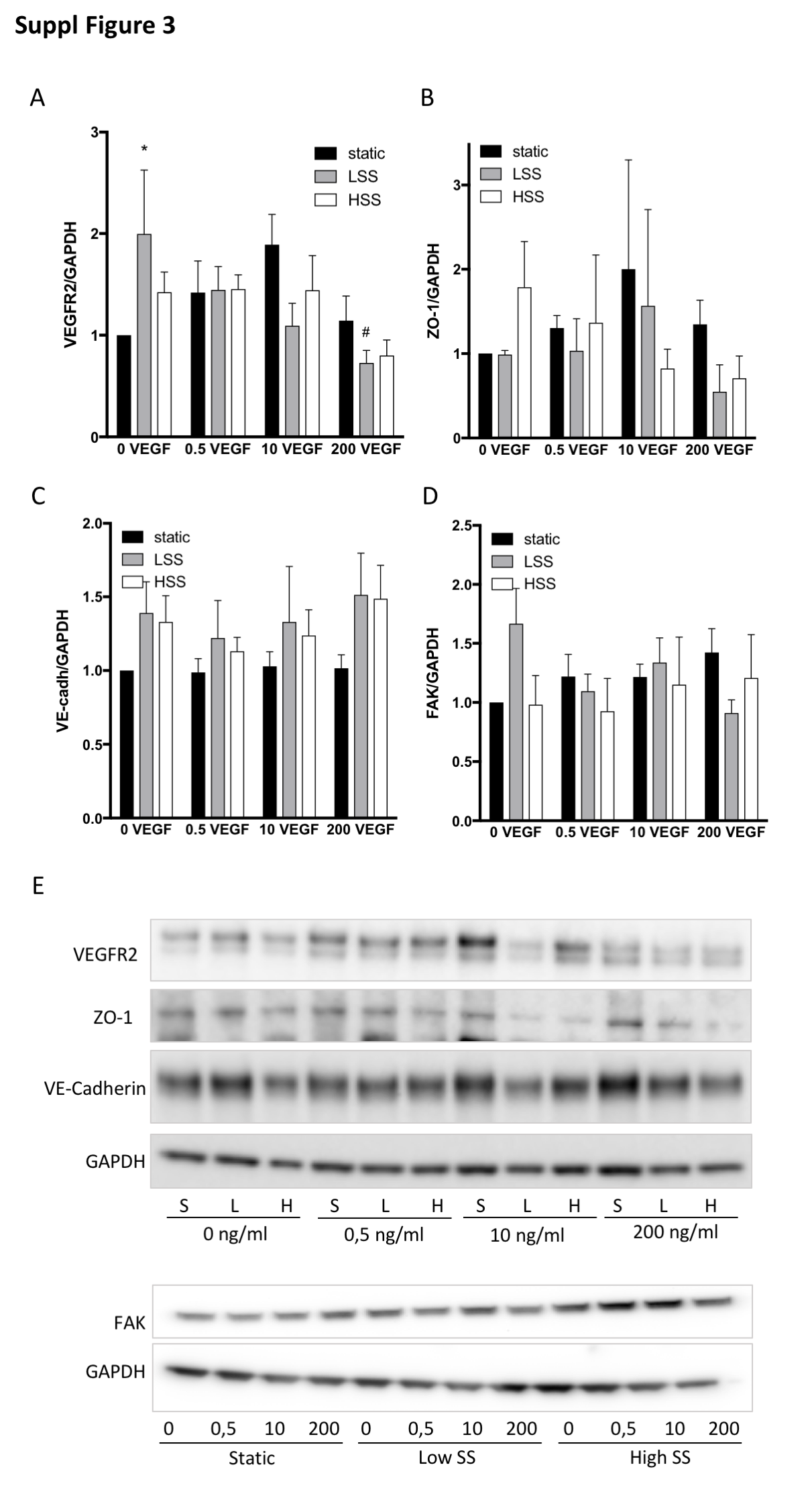

Supplement: Supplementary Figure 3 — Proteins expression upon flow and VEGF treatment. (A) VEGFR2 expression assessed by WB, N = 6. (B) ZO1 expression assessed by WB, N = 3. (C) VE-cadherin expression assessed by WB, N = 5. (D) FAK expression assessed by WB, N = 5. ANOVA followed by Tukey post Hoc; ∗p < 0.05; ∗∗p < 0.01. (E) Representative pictures of the quantified WB. [file Image_3.TIFF]

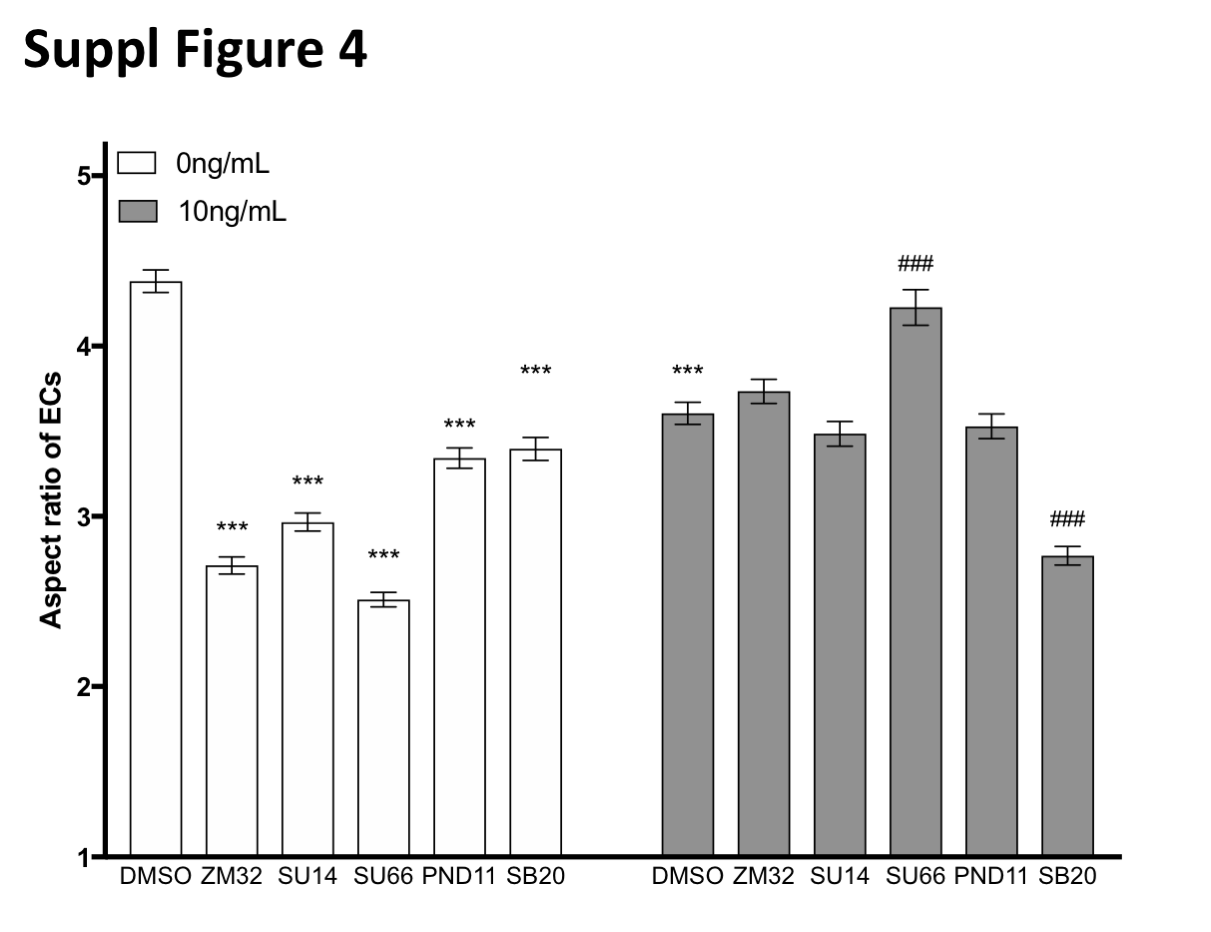

Supplement: Supplementary Figure 4 — Inhibitors effect on cell elongation. Quantification of aspect ratio (length of main axis/length of short axis) of endothelial cells under high SS with or without VEGF-A (10 ng/mL) and with or without inhibitors (DMSO, N = 5, inhibitors N = 3) One-way ANOVA; Tukey’s post hoc, ∗∗∗p < 0.001 compared to DMSO 0 VEGF; ###p < 0.001 compared to DMSO 10 VEGF. [file Image_4.TIFF]

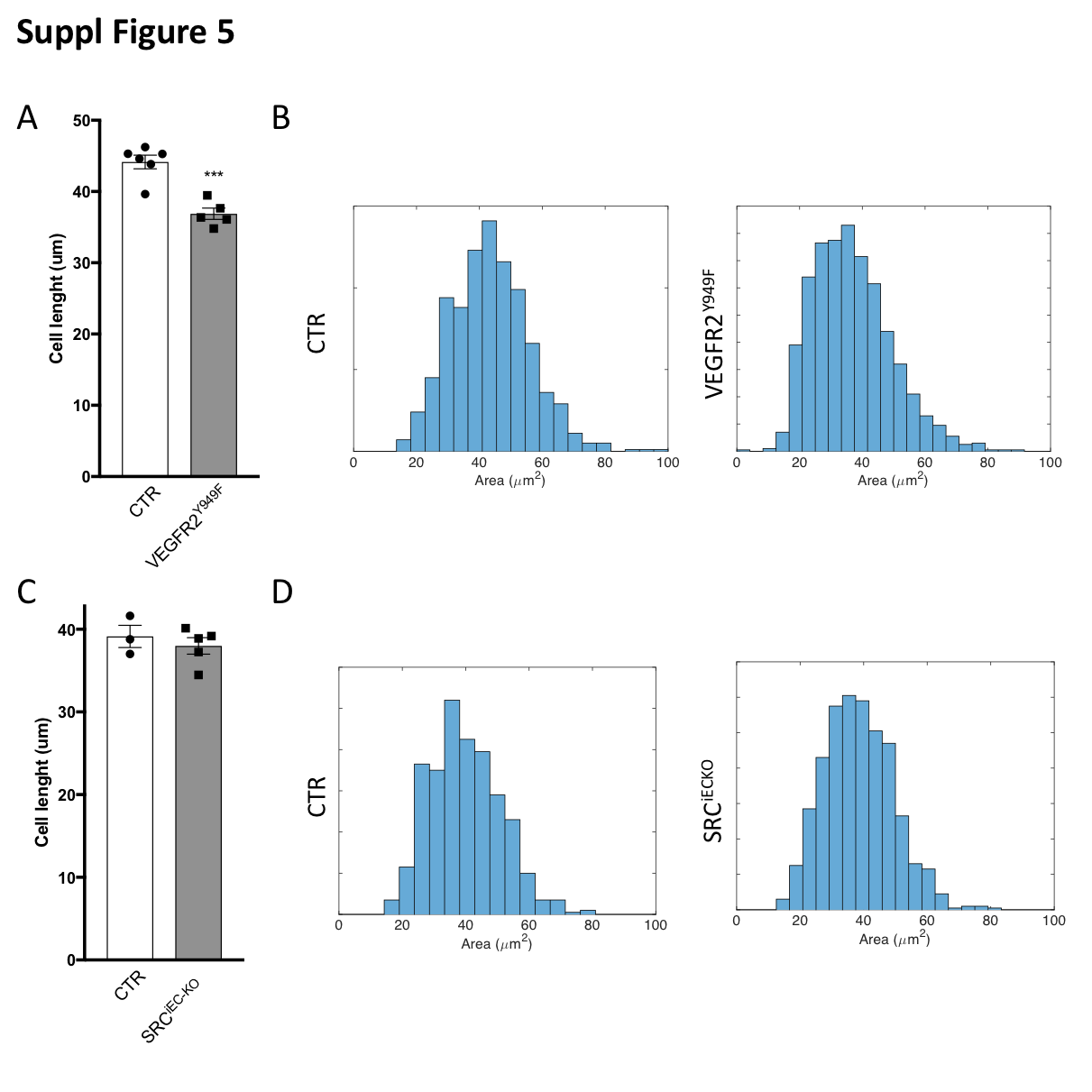

Supplement: Supplementary Figure 5 — VEGFR2 mutation impairs cell length but not SRC deletion in vivo. (A) Quantification of ECs length in the aortas of P6 pups littermate (CTR) or carrying VEGFR2 mutation (Y949F). N = 5 (B) Distribution of cell length. N = 5 (C) Quantification of ECs length in the aortas of P6 pups littermate (CTR) or deleted for SRC in ECs (SRCiEC–KO). N = 3–5 (D) Distribution of cell length. Unpaired T-Test; ∗∗∗p < 0.001. [file Image_5.TIFF]

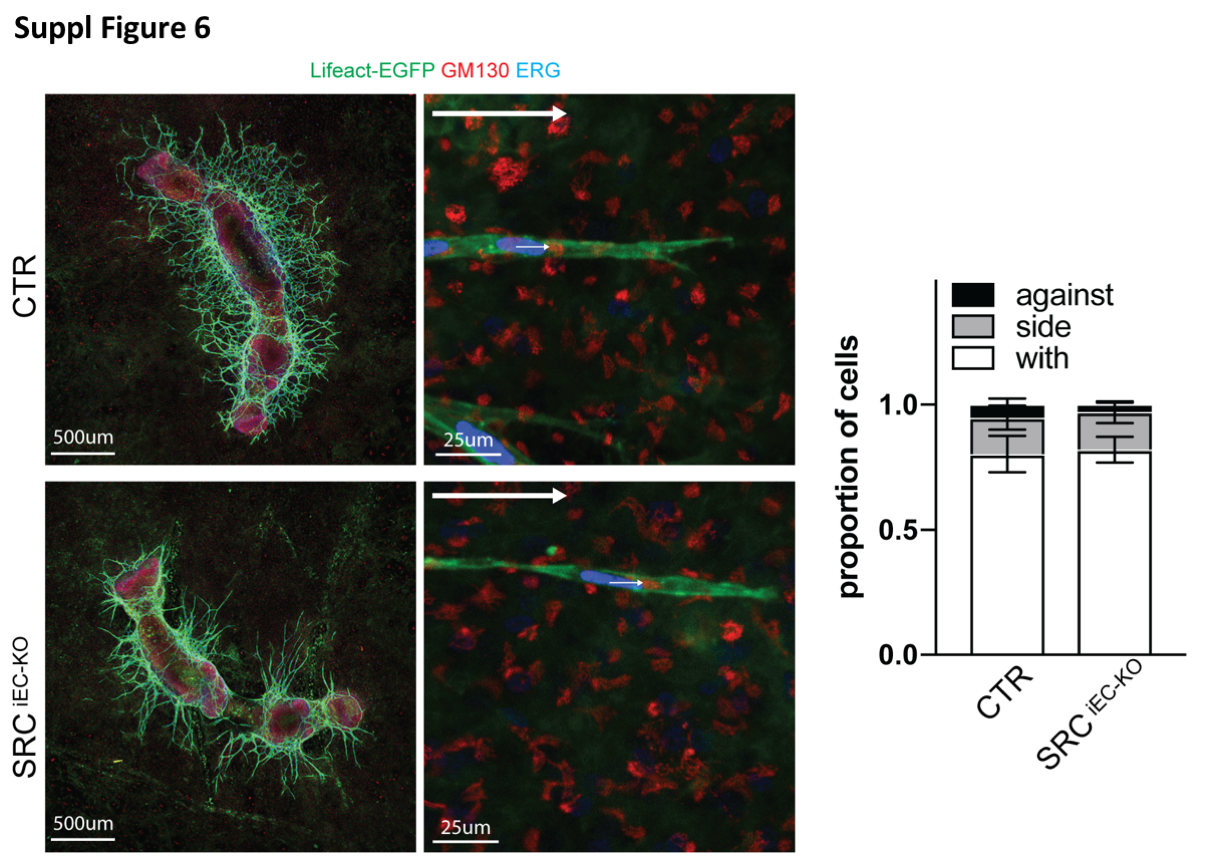

Supplement: Supplementary Figure 6 — ECs polarity is not impaired during directional sprouting upon loss of c-Src. Representative images and quantification of polarity of ECs sprouting out of metatarsal ex vivo. With means that ECs polarize in the direction of the sprout (0–30°); side, ECs present their golgi on the side of the cell (30–150°); against, ECs polarize in the opposite direction of the sprout (150–180°). N = 294 cells analyzed from 12 metatarsals from 2 independent experiments. [file Image_6.TIFF]
