# Supplementary material for: Endothelial Cell Orientation and Polarity Are Controlled by Shear Stress and VEGF Through Distinct Signaling Pathways
Source: Front Physiol. 2021 Mar 2;11:623769. doi: 10.3389/fphys.2020.623769 (PMC7960671; doi:10.3389/fphys.2020.623769)
Supplement: Supplementary Table 1 — p-Values for each comparison for Figures 2B,D. Two-way ANOVA; Tukey’s post hoc, ∗p < 0.05; ∗∗p < 0.01; ∗∗∗p < 0.001. Gray lines: statistics presented on the graphs. Bold: significant difference. [file Table_1.pdf]

Suppl Table 1

	Alignment		Polarity	
		Adjusted P Value		Adjusted P Value
S 0 vs. S 0,5	ns	>0,9999	ns	>0,9999
S 0 vs. S 10	ns	>0,9999	ns	>0,9999
S 0 vs. S 200	ns	0,9985	ns	0,9365
S 0 vs. L 0	ns	0,9996	***	**0,0003**
S 0 vs. L 0,5	ns	0,1403	ns	0,0599
S 0 vs. L 10	ns	>0,9999	ns	0,9989
S 0 vs. L 200	ns	0,1884	ns	>0,9999
S 0 vs. H 0	ns	0,1885	ns	>0,9999
S 0 vs. H 0,5	ns	0,3268	***	**0,0005**
S 0 vs. H 10	ns	>0,9999	*	**0,0119**
S 0 vs. H 200	****	**<0,0001**	ns	0,0824
S 0,5 vs. S 10	ns	>0,9999	ns	>0,9999
S 0,5 vs. S 200	ns	0,992	ns	0,9148
S 0,5 vs. L 0	ns	0,9969	***	**0,0002**
S 0,5 vs. L 0,5	ns	0,2112	*	**0,0496**
S 0,5 vs. L 10	ns	>0,9999	ns	0,9995
S 0,5 vs. L 200	ns	0,1237	ns	>0,9999
S 0,5 vs. H 0	ns	0,1237	ns	>0,9999
S 0,5 vs. H 0,5	ns	0,4454	***	**0,0007**
S 0,5 vs. H 10	ns	>0,9999	*	**0,0148**
S 0,5 vs. H 200	****	**<0,0001**	ns	0,0981
S 10 vs. S 200	ns	0,9995	ns	0,8855
S 10 vs. L 0	ns	0,9999	***	**0,0002**
S 10 vs. L 0,5	ns	0,1117	*	**0,0399**
S 10 vs. L 10	ns	>0,9999	ns	0,9998
S 10 vs. L 200	ns	0,2306	ns	>0,9999
S 10 vs. H 0	ns	0,2307	ns	>0,9999
S 10 vs. H 0,5	ns	0,2731	***	**0,0009**
S 10 vs. H 10	ns	>0,9999	*	**0,0188**
S 10 vs. H 200	****	**<0,0001**	ns	0,1185
S 200 vs. L 0	ns	>0,9999	*	**0,0407**
S 200 vs. L 0,5	*	**0,013**	ns	0,8055
S 200 vs. L 10	ns	0,9615	ns	0,4204
S 200 vs. L 200	ns	0,7331	ns	0,6179
S 200 vs. H 0	ns	0,7332	ns	0,5662
S 200 vs. H 0,5	*	**0,0429**	****	**<0,0001**
S 200 vs. H 10	ns	0,9901	****	**<0,0001**
S 200 vs. H 200	***	**0,0005**	***	**0,0007**
L 0 vs. L 0,5	*	**0,0184**	ns	0,8881
L 0 vs. L 10	ns	0,9803	****	**<0,0001**
L 0 vs. L 200	ns	0,6549	****	**<0,0001**
L 0 vs. H 0	ns	0,6551	****	**<0,0001**
L 0 vs. H 0,5	ns	0,0586	****	**<0,0001**
L 0 vs. H 10	ns	0,9961	****	**<0,0001**
L 0 vs. H 200	***	**0,0003**	****	**<0,0001**
L 0,5 vs. L 10	ns	0,3387	**	**0,0039**
L 0,5 vs. L 200	****	**<0,0001**	*	**0,01**
L 0,5 vs. H 0	****	**<0,0001**	**	**0,0079**
L 0,5 vs. H 0,5	ns	>0,9999	****	**<0,0001**
L 0,5 vs. H 10	ns	0,2236	****	**<0,0001**
L 0,5 vs. H 200	****	**<0,0001**	****	**<0,0001**
L 10 vs. L 200	ns	0,0673	ns	>0,9999
L 10 vs. H 0	ns	0,0673	ns	>0,9999
L 10 vs. H 0,5	ns	0,618	*	**0,0119**
L 10 vs. H 10	ns	>0,9999	ns	0,1406
L 10 vs. H 200	****	**<0,0001**	ns	0,495
L 200 vs. H 0	ns	>0,9999	ns	>0,9999
L 200 vs. H 0,5	***	**0,0001**	**	**0,0047**
L 200 vs. H 10	ns	0,1158	ns	0,0693
L 200 vs. H 200	ns	0,1261	ns	0,3124
H 0 vs. H 0,5	***	**0,0001**	**	**0,006**
H 0 vs. H 10	ns	0,1159	ns	0,0839
H 0 vs. H 200	ns	0,126	ns	0,3558
H 0,5 vs. H 10	ns	0,4642	ns	0,9989
H 0,5 vs. H 200	****	**<0,0001**	ns	0,9102
H 10 vs. H 200	****	**<0,0001**	ns	>0,9999
