# Supplementary material for: Endothelial Cell Orientation and Polarity Are Controlled by Shear Stress and VEGF Through Distinct Signaling Pathways
Source: Front Physiol. 2021 Mar 2;11:623769. doi: 10.3389/fphys.2020.623769 (PMC7960671; doi:10.3389/fphys.2020.623769)
Supplement: Supplementary Table 2 — p-Values for each comparison for Figures 3C–F. Two-way ANOVA; Fisher LSD. ∗p < 0.05; ∗∗p < 0.01; ∗∗∗p < 0.001. Gray lines: statistics presented on the graphs. Bold: significant difference. [file Table_2.pdf]

Suppl Table 2	VE-cadh 685/VE-cadh		VEGFR2 951 / GAPDH		VEGFR2 1175 / GAPDH		PXL 118 / PXL	
		Individual P Value		Individual P Value		Individual P Value		Individual P Value
0:static vs. 0:Low SS	***	**0,0007**	ns	0,4289	ns	0,5059	ns	0,4202
0:static vs. 0:High SS	ns	0,3814	ns	0,6037	ns	0,1234	ns	0,2872
0:static vs. 0,5:static	*	**0,0428**	ns	0,4307	ns	0,8245	ns	0,7449
0:static vs. 0,5:Low SS	****	**<0,0001**	ns	0,0798	ns	0,2233	ns	0,0797
0:static vs. 0,5:High SS	*	0,0263	ns	0,2753	ns	0,4337	ns	0,0718
0:static vs. 10:static	***	**0,0001**	ns	0,4354	ns	0,2869	ns	0,931
0:static vs. 10:Low SS	***	**0,0003**	*	**0,0402**	***	**0,0003**	ns	0,0785
0:static vs. 10:High SS	ns	0,3094	ns	0,197	ns	0,0806	**	**0,0021**
0:static vs. 200:static	***	**0,0008**	**	**0,0016**	***	**0,0004**	ns	0,2844
0:static vs. 200:Low SS	*	**0,0263**	***	**0,0002**	****	**<0,0001**	*	**0,0477**
0:static vs. 200:High SS	ns	0,6743	ns	0,0754	***	**0,0001**	****	**<0,0001**
0:Low SS vs. 0:High SS	**	**0,0062**	ns	0,7838	ns	0,371	ns	0,7936
0:Low SS vs. 0,5:static	ns	0,092	ns	0,9975	ns	0,6565	ns	0,6293
0:Low SS vs. 0,5:Low SS	ns	0,1451	ns	0,3228	ns	0,574	ns	0,3333
0:Low SS vs. 0,5:High SS	ns	0,1403	ns	0,7602	ns	0,9053	ns	0,3092
0:Low SS vs. 10:static	ns	0,492	ns	0,991	ns	0,6849	ns	0,4713
0:Low SS vs. 10:Low SS	ns	0,7859	ns	0,1925	**	**0,0022**	ns	0,3296
0:Low SS vs. 10:High SS	**	**0,0087**	ns	0,6101	ns	0,2676	*	**0,0185**
0:Low SS vs. 200:static	ns	0,9622	*	**0,0131**	**	**0,0024**	ns	0,7888
0:Low SS vs. 200:Low SS	ns	0,14	**	**0,0015**	****	**<0,0001**	ns	0,2288
0:Low SS vs. 200:High SS	***	**0,0002**	ns	0,3097	***	0,0008	***	**0,0004**
0:High SS vs. 0,5:static	ns	0,2244	ns	0,7862	ns	0,1841	ns	0,4576
0:High SS vs. 0,5:Low SS	***	**0,0001**	ns	0,2091	ns	0,7369	ns	0,4785
0:High SS vs. 0,5:High SS	ns	0,1528	ns	0,5628	ns	0,437	ns	0,4482
0:High SS vs. 10:static	**	**0,0011**	ns	0,7924	ns	0,6224	ns	0,3275
0:High SS vs. 10:Low SS	**	**0,0032**	ns	0,1173	*	**0,022**	ns	0,4738
0:High SS vs. 10:High SS	ns	0,8845	ns	0,4342	ns	0,827	*	**0,0345**
0:High SS vs. 200:static	**	**0,0069**	**	**0,0065**	*	**0,0237**	ns	0,995
0:High SS vs. 200:Low SS	ns	0,1531	***	**0,0007**	****	**<0,0001**	ns	0,3439
0:High SS vs. 200:High SS	ns	0,2002	ns	0,1995	**	**0,009**	***	**0,0009**
0,5:static vs. 0,5:Low SS	**	**0,0033**	ns	0,3213	ns	0,3165	ns	0,1499
0,5:static vs. 0,5:High SS	ns	0,8203	ns	0,7578	ns	0,5734	ns	0,1367
0,5:static vs. 10:static	*	**0,0218**	ns	0,9936	ns	0,3968	ns	0,8111
0,5:static vs. 10:Low SS	ns	0,0536	ns	0,1914	***	**0,0006**	ns	0,1479
0,5:static vs. 10:High SS	ns	0,2821	ns	0,6079	ns	0,1241	**	**0,0052**
0,5:static vs. 200:static	ns	0,1007	*	**0,013**	***	**0,0007**	ns	0,4538
0,5:static vs. 200:Low SS	ns	0,8213	**	**0,0015**	****	**<0,0001**	ns	0,0947
0,5:static vs. 200:High SS	*	**0,017**	ns	0,3082	***	**0,0002**	****	**<0,0001**
0,5:Low SS vs. 0,5:High SS	**	**0,0058**	ns	0,4916	ns	0,6572	ns	0,9601
0,5:Low SS vs. 10:static	ns	0,4269	ns	0,3174	ns	0,8752	ns	0,0949
0,5:Low SS vs. 10:Low SS	ns	0,2301	ns	0,7465	**	**0,0097**	ns	0,994
0,5:Low SS vs. 10:High SS	***	**0,0002**	ns	0,6285	ns	0,5798	ns	0,1504
0,5:Low SS vs. 200:static	ns	0,1333	ns	0,1164	*	**0,0105**	ns	0,4824
0,5:Low SS vs. 200:Low SS	**	**0,0057**	*	**0,0203**	****	**<0,0001**	ns	0,8101
0,5:Low SS vs. 200:High SS	****	**<0,0001**	ns	0,9779	**	**0,0037**	**	**0,0072**
0,5:High SS vs. 10:static	*	**0,0359**	ns	0,7516	ns	0,774	ns	0,0858
0,5:High SS vs. 10:Low SS	ns	0,0845	ns	0,3143	**	**0,003**	ns	0,9661
0,5:High SS vs. 10:High SS	ns	0,196	ns	0,8373	ns	0,321	ns	0,1646
0,5:High SS vs. 200:static	ns	0,1526	*	**0,0271**	**	**0,0033**	ns	0,452
0,5:High SS vs. 200:Low SS	ns	0,999	**	**0,0035**	****	**<0,0001**	ns	0,8491
0,5:High SS vs. 200:High SS	*	**0,0101**	ns	0,4744	**	**0,0011**	**	0,0082
10:static vs. 10:Low SS	ns	0,676	ns	0,1888	**	**0,0065**	ns	0,0935
10:static vs. 10:High SS	**	**0,0016**	ns	0,6023	ns	0,478	**	**0,0027**
10:static vs. 200:static	ns	0,4631	*	**0,0127**	**	**0,007**	ns	0,3245
10:static vs. 200:Low SS	*	**0,0358**	**	**0,0015**	****	**<0,0001**	ns	0,0576
10:static vs. 200:High SS	****	**<0,0001**	ns	0,3045	**	**0,0024**	****	<0,0001
10:Low SS vs. 10:High SS	**	**0,0045**	ns	0,4211	*	**0,0364**	ns	0,1524
10:Low SS vs. 200:static	ns	0,7498	ns	0,2077	ns	0,9743	ns	0,4777
10:Low SS vs. 200:Low SS	ns	0,0843	*	**0,0426**	**	**0,0053**	ns	0,816
10:Low SS vs. 200:High SS	***	**0,0001**	ns	0,7676	ns	0,7146	**	0,0073
10:High SS vs. 200:static	**	**0,0098**	*	**0,0431**	*	**0,0391**	*	0,035
10:High SS vs. 200:Low SS	ns	0,1965	**	**0,0061**	****	**<0,0001**	ns	0,2284
10:High SS vs. 200:High SS	ns	0,1562	ns	0,609	*	**0,0155**	ns	0,1841
200:static vs. 200:Low SS	ns	0,1523	ns	0,418	**	**0,0049**	ns	0,3471
200:static vs. 200:High SS	***	**0,0003**	ns	0,1227	ns	0,6907	***	**0,001**
200:Low SS vs. 200:High SS	*	**0,0101**	*	**0,0217**	*	**0,0135**	*	**0,0134**
